# Optimized bio-signal reconstruction and watermarking via enhanced fractional orthogonal moments

**DOI:** 10.1038/s41598-025-16086-2

**Published:** 2025-08-19

**Authors:** Gaber Hassan, Khalid M. Hosny, Islam S. Fathi

**Affiliations:** 1 Department of Computer Science, Faculty of computers and information, Arish University, Al-Arish, 45511 Egypt; 2 Department of Information Technology, Faculty of Computers and Informatics, Zagazig University, 44519 Zagazig, Egypt; 3 Department of Computer Science, Faculty of Information Technology, Ajloun National University, P.O.43, Ajloun, 26810 Jordan

**Keywords:** Bio-signals watermarking, Bio-signals reconstruction, Fractional tchebichef moments, Schwarz-Rutishauser, Internet of healthcare things, Engineering, Mathematics and computing

## Abstract

Orthogonal Tchebichef moments of fractional order (FrTMs) serve as descriptors for signals and images. Many fields, including signal analysis and watermarking, have relied heavily on such moments. This study addresses three critical limitations in existing approaches: the computational burden of higher-order moment calculations, numerical instability affecting reconstruction accuracy, and orthogonality deterioration in large-scale signal processing. Furthermore, using the QR decomposition approach is crucial to maintain the orthogonality of the higher-order moments. We introduce an improved computational framework with three main scientific contributions as development of an optimized set of three interrelated second-order recurrence equations for normalized FrTMs, implementation of the Schwarz-Rutishauser algorithm as an alternative to classical QR decomposition methods, maintaining orthogonality with substantially lower computational overhead; and integration of these innovations into a comprehensive system for biomedical signal reconstruction and watermarking. The method in question was tested on two benchmark datasets the MIT-BIH arrhythmia and CHB-MIT Scalp EEG. The findings indicate that the proposed methodology exhibits significantly higher performance levels than current methodologies, with a 64.3% improvement in PSNR (reaching 147.08 dB compared to 89.74 dB in existing approaches), 89.7% reduction in MSE (0.0092 versus 0.09 average), and 84.1% decrease in bit error rate (0.25 versus 1.57) for watermarking applications. Processing time was also reduced by 64.3% compared to competing methods, making this approach substantially more efficient for implementation in Internet of Healthcare Things (IoHT) contexts.

## Introduction

Using mathematical tools for characterizing objects in different dimensions (i.e., 1-D, 2-D, and 3-D objects) has become one of the most critical research fields researchers deal with in computer science. Moments are one of the most important mathematical tools researchers have greatly appreciated since the last century. Mathematically, moments can be defined as projecting an object function onto a set of basis functions. The projection above yields several necessary statistical measures in describing the object^1^. Orthogonal Moments (OMs) are characterized by orthogonal basis functions in polar coordinates^2^ or Cartesian coordinates space^3^.

Orthogonality, is a mathematical property where the basis functions used to compute the moments are independent and non-overlapping in their representation of information. Orthogonal functions guarantee that every moment records a distinct characteristic of the image or signal, much like perpendicular vectors in geometry, which point in different directions and have no shared component. According to this crucial property, orthogonal moments are widely used in various applications of image and signal analysis. These include signal and image reconstruction^4^. They’re also used to embed hidden watermarks in images (i.e., image watermarking)^5^ and to detect copy-move in color images^6^. These techniques support encryption of both gray-scale and color images making then more secure^7^-^8^. In the medical field they used in many applications as biomedical image retrieval^9^. They are valuable in image representation and recognition^10^. They create invariant features that help computers identify images correctly^11^. Additionally, they allow efficient compression of volumetric medical images^12^. These mathematical methods also support face recognition^13^, color image steganography^14^. Beyond images, they used with biomedical signals in many applications such as, compression of bio-signals^15^. Finally, they play a role in systems that monitor patients’ health from a distance (i.e., Remote Healthcare Monitoring Systems)^16^.

Fractional order refers to derivatives or integrals of non-integer order. Unlike the usual calculus which deals with whole numbers, fractional calculus extends this to any real or complex number. Fractional-order polynomials (or polynomials with non-integer exponents) are important because they generalize classical polynomial frameworks, enabling more flexible and accurate modeling of complex real-world phenomena. Recently, a novel collection of fractional-order polynomials has been formulated. These include fractional-order Legendre-Fourier moments^17^, fractional-order generic Jacobi-Fourier moments^18^, fractional-order polar harmonic transforms^19^, Fractional-order orthogonal Tchebichef moments^20^, and fractional-order Charlier moments^21^. Motivated by mathematicians’ achievements, researchers have formulated fractional-order variations. Scholars have increasingly turned to utilizing these fractional moments across several research applications.

Research shows that fractional Discrete Orthogonal Moments (DOMs) offer benefits in many applications. However, they face several challenges: higher-order moments can become numerically unstable when basis function values fluctuate, traditional polynomial calculations are time-consuming, and error accumulation in complex signals can weaken orthogonal properties. To address these problems, researchers have developed several solutions: they used weighted fractional Tchebichef polynomials to improve stability, created a normalized formula to calculate results faster, and applied mathematical techniques like Gram-Schmidt Method (GSM), Householder Method (HM), and Given Rotations Methods (GRM)^22^ to preserve data integrity.

This research introduces an innovative bio-signal reconstruction and watermarking algorithm, integrating fractional-order Tchebichef moments (FrTMs) with the Schwarz-Rutishauser method.We developed three second-order recurrence formulas for normalized FrTMs that offer substantial computational benefits. The selection of the Schwarz-Rutishauser method as an alternative to traditional QR decomposition techniques was motivated by several critical factors inherent to bio-signal processing applications. Classical QR decomposition methods such as Gram-Schmidt (GSM), Householder (HM), and Givens Rotations (GRM) have been widely employed in orthogonalization processes. However, these methods present significant limitations when processing high-dimensional bio-signals, particularly at higher orders of polynomial basis functions. The primary motivation stems from the numerical instability that plagues traditional orthogonalization approaches when computing high-order fractional moments. When analyzing complex bio-signals such as ECG and EEG data, the traditional Gram-Schmidt method suffers from error propagation and loss of orthogonality, particularly as the order of the polynomials increases. This numerical degradation directly compromises the quality of reconstructed signals, which is unacceptable in medical applications where precision is paramount. The Schwarz-Rutishauser algorithm represents a mathematically elegant solution to these challenges. Unlike the classical Gram-Schmidt process, which requires the explicit computation and storage of intermediate orthogonal vectors, the Schwarz-Rutishauser method employs an optimized recursive approach that maintains orthogonality throughout the computation process. This method essentially reformulates the orthogonalization procedure to minimize the accumulation of rounding errors that typically occur in floating-point arithmetic operations. Computational efficiency represents another crucial motivation for adopting the Schwarz-Rutishauser approach. When processing large-scale bio-signals, especially in resource-constrained environments like IoHT devices, the computational complexity of O(mn²) offered by the Schwarz-Rutishauser algorithm provides a significant advantage over traditional QR decomposition methods. This n-fold reduction in computational complexity directly translates to faster processing times without sacrificing accuracy, making it particularly suitable for real-time monitoring applications where prompt analysis of physiological signals is essential.

Our research introduces a pioneering set of three interrelated second-order recurrence equations specifically engineered for normalized Fractional Tchebichef Moments (FrTMs). Unlike traditional approaches that suffer from computational inefficiency and numerical instability at higher orders, our formulation provides a mathematically elegant solution that reduces computational complexity while maintaining numerical precision. This improvement represents a fundamental mathematical advancement in orthogonal moment computation, particularly beneficial for analyzing complex biomedical signals.

The present work aims to present the following contributions:

- Introduces an improved set of three interrelated second-order recurrence equations designed to work with normalized Fractional Tchebichef Moments (FrTMs).
- Implements the Schwarz-Rutishauser technique, an innovative approach to QR decomposition that enhances numerical stability while reducing computational overhead compared to conventional methods.
- Presents a high-performance algorithm for efficiently reconstructing biomedical signals while maintaining signal integrity.
- Developed a computationally optimized algorithm designed explicitly for accurately watermarking biomedical signal data.

The paper is structured in seven sections. Following the introduction, in Sect. 2 literature review is presented. Section 3 introduces the fundamental concepts of FrTMs. Section 3 examines the mathematical foundations and calculations involved in fractional Tchebichef polynomials (FrTP). Section 4 explores the innovative Schwarz-Rutishauser methodology. Section 5 provides an in-depth analysis of our proposed FrTM algorithm, incorporating the normalized FrTP three-term second-order recurrence formula and the Schwarz-Rutishauser method for signal reconstruction and watermarking applications. Section 6 presents our experiments and analysis, and Sect. 7 summarizes our conclusions and insights.

## Literature review

In an earlier work, Hamidi et al.^23^ investigated a hybrid blind robust image watermarking technique based on Discrete Fourier Transform (DFT), Discrete Cosine Transform (DCT), and Arnold transform. Their approach showed particular promise for applications requiring high security and robustness. Hamidi et al.^24^ developed a hybrid robust image watermarking method based on DWT-DCT and Scale-Invariant Feature Transform (SIFT) for copyright protection. Their approach leveraged the complementary strengths of different transform domains to create a watermarking technique that was both imperceptible and robust. The experimental results demonstrated strong resistance against various attacks, including compression, noise addition, and geometric transformations, while maintaining visual quality. Hasan et al.^25^ proposed an encryption-based image watermarking algorithm in the 2D-DWT-DCT domains. Their approach incorporated cryptographic techniques to enhance the security of the watermarking process.

Awasthi and Srivastava^26^ proposed a multiple image watermarking scheme with dual authentication designed specifically for smart city environments. Their framework utilized advanced cryptographic techniques combined with transform domain methods to ensure that medical images maintain their diagnostic value while being securely transmitted. Building on this research, Awasthi and Srivastava^27^ further refined their approach by developing an optimized and secured image watermarking technique with dual authentication specifically tailored for the Internet of Medical Things. Their method combined discrete wavelet transform (DWT) with spatial domain techniques to embed watermarks that could withstand various attacks while preserving the clinical quality of medical images.

Al-sudani et al.^28^ presented a multithreading-based algorithm for high-performance Tchebichef polynomials computation with higher orders. Their work addressed one of the primary challenges in applying orthogonal moment-based techniques to large medical datasets: computational complexity. By leveraging parallel processing, the authors achieved significant reductions in computation time (up to 80% in some cases) without sacrificing accuracy. Mohammed et al.^29^ explored the application of orthogonal polynomials for three-dimensional object recognition using an embedded kernel approach. While not directly focused on bio-signals, their work demonstrates the versatility of orthogonal moments in capturing complex spatial relationships, which has important implications for volumetric medical data analysis. Radeaf et al.^30^ proposed a steganography technique based on orthogonal moments. Their approach utilized the properties of orthogonal transformations to embed information in a way that minimizes distortion of the cover image while maximizing resistance to detection. Recent studies^31–33^ have begun exploring the integration of fractional-order orthogonal moments with deep learning techniques for enhanced watermarking and reconstruction performance. Also, the software deformation prediction based on neural networks and feature selection These approaches leverage the flexibility offered by fractional-order parameters to adaptively optimize the tradeoff between robustness and imperceptibility based on the specific characteristics of different bio-signals. Naser et al.^34^ investigated the improvement of cardiovascular prediction performance using machine learning-based feature selection. Their work incorporated orthogonal moment-based features to capture subtle patterns in cardiovascular signals that might be missed by conventional time-domain analysis. Idan et al.^35^ demonstrated the application of separable moments for fast shot boundary detection, combined with support vector machines for classification. Although their primary application was in video processing, the technique’s ability to efficiently detect abrupt changes in signal patterns has potential applications in bio-signal analysis, particularly for identifying anomalies or transitions between different physiological states.

This research introduces an innovative bio-signal reconstruction and watermarking algorithm, integrating fractional-order Tchebichef moments (FrTMs) with the Schwarz-Rutishauser method^36^. We developed three second-order recurrence formulas for normalized FrTMs that offer substantial computational benefits. The Schwarz-Rutishauser algorithm proved valuable in preserving orthogonality when analyzing extensive bio-signals at higher orders. While our team has experience with conventional QR Decomposition approaches (Gram-Schmidt, Householder, and Given Rotations methods), testing revealed these techniques significantly increased processing time, creating inefficiencies for Internet of Health Things (IoHT) devices. The Schwarz-Rutishauser method is a numerically stable algorithm for orthogonalizing vectors while minimizing errors caused by rounding or ill-conditioned data. It’s an iterative refinement of Gram-Schmidt, designed to improve numerical stability. Its primary goal is to preserve data integrity, this ensures the preservation of the orthogonality at higher orders and when working with noisy or nearly dependent datasets. The Schwarz-Rutishauser approach, an enhancement of the traditional Gram-Schmidt method, offers improved efficiency with its $\:O\left({n}^{2}\right)$ Computational complexity is notably lower than that of classical QR Decomposition methods. To verify the effectiveness of combining FrTMs with the Schwarz-Rutishauser method, we analyzed ECG and EEG signals from the MIT-BIH arrhythmia and CHB-MIT datasets. Empirical testing of signal reconstruction and watermarking demonstrated marked performance improvements.

## Fractional order discrete Tchebichef moments

The 1D Fractional orders Tchebichef orthogonal moments$\:{\widehat{\:{\text{F}}_{\text{r}}\text{T}\text{M}}}_{n}$ with the order, $\:n$ can be mathematically formulated with the Fractional order Tchebichef Polynomials $\:{\stackrel{\sim}{{F}_{\text{r}}T}}_{n}^{\alpha\:}\left(t\right)\:\:$as follows:

1 $$\:{\widehat{\:{\text{F}}_{\text{r}}\text{T}\text{M}}}_{n}=\:\sum_{x=0}^{N-1}\:{\stackrel{\sim}{{F}_{\text{r}}T}}_{n}^{\alpha\:}\left(t\right)\:s\left(x\right),\:\:\:\:\:\:\:\:\:\:n=\text{0,1},2,\dots\:,N-1.$$

where $\:s\left(x\right)$ represents a signal of size 1×N.

The reconstructed signal $\:\widehat{\text{s}}\left(\text{x}\right)\:$can be expressed from the inverse transformation of Fractional Tchebichef Moments.

2 $$\:\widehat{\text{s}}\left(\text{x}\right)=\:\sum_{n=0}^{{\text{n}}_{\text{m}\text{a}\text{x}}}{\widehat{\:{\text{F}}_{\text{r}}\text{T}\text{M}}}_{n}\:{\stackrel{\sim}{{F}_{\text{r}}T}}_{n}^{\alpha\:}\left(t\right)\:,\:\:\:\:\:\:\:\:\:\:x=\text{0,1},2,\dots\:,N-1.$$

where $\:{\widehat{\:{\text{F}}_{\text{r}}\text{T}\text{M}}}_{n}\:and\:\:{\stackrel{\sim}{{F}_{\text{r}}T}}_{n}^{\alpha\:}\left(t\right)\:\:$Fractional order Tchebichef moments and Polynomials, respectively.

## Improved computational methods for Fractional-Order Tchebichef polynomials

The Fractional order Tchebichef polynomials $\:{{F}_{\text{r}}T}_{n}^{\propto\:}\left(t\right)$ defined as^37^:

$$\:{{F}_{\text{r}}\text{T}}_{\text{n}}^{\propto\:}\left(\text{t}\right)={\text{T}}_{\text{n}}\left(1-2{\text{t}}^{\propto\:}\right),\:\text{w}\text{h}\text{e}\text{r}\text{e}\:\text{t}\in\:[0,\:1]\:\text{a}\text{n}\text{d}\:\propto\:\:>0$$

The analytical form of $\:{{F}_{\text{r}}T}_{1}^{\propto\:}\left(t\right)$ obtained as

3 $$\:{{F}_{\text{r}}T}_{n}^{\propto\:}\left(t\right)=\:\sum_{k=0}^{n}{(-1)}^{k}\frac{n{2}^{2k}\left(n+k-1\right)!}{\left(n-k\right)!\left(2k\right)!}{t}^{\propto\:k}=\sum_{k=0}^{n}{\beta\:}_{n,k}{t}^{\alpha\:k},$$

where

$$\:{\beta\:}_{n,k}={(-1)}^{k}\frac{n{2}^{2k}\left(n+k-1\right)!}{\left(n-k\right)!\left(2k\right)!}\:\:\:\:\:\:\:\:\text{a}\text{n}\text{d}\:\:\:\:\:\:\:\:\:\:\:\:{\beta\:}_{0,k}=1.$$

$\:{{F}_{\text{r}}T}_{n}^{\propto\:}\left(t\right)$ can calculated rapidly based on the following three terms: recurrence formula

$$\:{{F}_{\text{r}}T}_{0}^{\propto\:}\left(t\right)=1,$$

$$\:{{F}_{\text{r}}T}_{1}^{\propto\:}\left(t\right)=1-2{t}^{\propto\:},$$

4 $$\:{{F}_{\text{r}}T}_{n+1}^{\propto\:}\left(t\right)=\left(2-4{t}^{\propto\:}\right){FT}_{n}^{\propto\:}\left(t\right)-{FT}_{n-1}^{\propto\:}\left(t\right).\:\:\:\:\:\:\:\:n=1,\:2,\:\dots\:,\:N-1.$$

The weighted Fractional order Tchebichef polynomials $\:{\stackrel{\sim}{{F}_{\text{r}}T}}_{n}^{\alpha\:}\left(t\right)$ defined as

5 $$\:{\stackrel{\sim}{{F}_{\text{r}}T}}_{n}^{\alpha\:}\left(t\right)=\sqrt{\frac{w\left(t\right)}{{c}_{n}}}{{F}_{\text{r}}T}_{n}^{\propto\:}\left(t\right),$$

where the weight function $\:w\left(t\right)$ is defined as

6 $$\:w\left(t\right)=\frac{{t}^{\frac{\propto\:}{2}-1}}{\sqrt{1-{t}^{\propto\:}}},$$

also $$\:{c}_{0}=2,\:and\:{c}_{n}=1\:for\:n\:\:1$$.

The normalized three-term recurrence relationship$\:\:{\stackrel{\sim}{{\text{F}}_{\text{r}}\text{T}}}_{\text{n}}^{{\upalpha\:}}\left(\text{t}\right)$ can be derived through the following process, beginning with establishing two fundamental initial conditions:

$$\:{\stackrel{\sim}{{F}_{\text{r}}T}}_{0}^{\alpha\:}\left(t\right)=\sqrt{\frac{w\left(t\right)}{{c}_{0}}}{{F}_{\text{r}}T}_{0}^{\propto\:}\left(t\right)$$

$$\:\text{w}\text{h}\text{e}\text{r}\text{e}\:{{F}_{\text{r}}T}_{0}^{\propto\:}\left(t\right)=1\:\text{a}\text{n}\text{d}\:{c}_{0}=2,\:\text{t}\text{h}\text{e}\text{n}$$

$$\:{\stackrel{\sim}{{F}_{\text{r}}T}}_{0}^{\alpha\:}\left(t\right)=\frac{1}{\sqrt{2}}\:\sqrt{w\left(t\right)}.\:\:$$

$$\:{\stackrel{\sim}{{F}_{\text{r}}T}}_{0}^{\alpha\:}\left(t\right)=\frac{1}{\sqrt{2}}\:\frac{\sqrt{{t}^{\frac{\propto\:}{2}-1}}}{1-{t}^{\propto\:}}$$

7 $$\:{\stackrel{\sim}{{F}_{\text{r}}T}}_{0}^{\alpha\:}\left(t\right)\:\:\:\:\:\:\:=\:\:\:\:\frac{1}{\sqrt{2}}\:\frac{{t}^{\frac{\propto\:}{4}-\frac{1}{2}}}{1-{t}^{\propto\:}}$$

Similarly,

$$\:{\stackrel{\sim}{{F}_{\text{r}}T}}_{1}^{\alpha\:}\left(t\right)=\sqrt{\frac{w\left(t\right)}{{c}_{1}}}{{F}_{\text{r}}T}_{1}^{\propto\:}\left(t\right),\:$$

$$\:\text{w}\text{h}\text{e}\text{r}\text{e}\:{{F}_{\text{r}}T}_{1}^{\propto\:}\left(t\right)=1-2{t}^{\propto\:}\:\text{a}\text{n}\text{d}\:{c}_{1}=1,\:\text{t}\text{h}\text{e}\text{n},$$

$$\:{\stackrel{\sim}{{F}_{\text{r}}T}}_{1}^{\alpha\:}\left(t\right)=\sqrt{w\left(t\right)}\:\:(1-2{t}^{\propto\:}),\:$$

8 $$\:{\stackrel{\sim}{{F}_{\text{r}}T}}_{1}^{\alpha\:}\left(t\right)=\frac{{t}^{\frac{\propto\:}{4}-\frac{1}{2}}}{1-{t}^{\propto\:}}\:(1-2{t}^{\propto\:}),\:$$

Following the established initial conditions, the generalized recurrence relationship can be expressed as.

Utilizing the three-term recurrence formula presented in Eq. (4) and for $\:n=1,\:2,\:\dots\:,\:N-1$:

9 $$\:{{F}_{\text{r}}T}_{n+1}^{\propto\:}\left(t\right)=\left(2-4{t}^{\propto\:}\right){{F}_{\text{r}}T}_{n}^{\propto\:}\left(t\right)-{{F}_{\text{r}}T}_{n-1}^{\propto\:}\left(t\right).$$

where,

$$\:{\stackrel{\sim}{{F}_{\text{r}}T}}_{n}^{\alpha\:}\left(t\right)=\sqrt{\frac{w\left(t\right)}{{c}_{n}}}{{F}_{\text{r}}T}_{n}^{\propto\:}\left(t\right),\:\:\:\:\:\:\:\:\:\:\:\:\:\:\:\:\:\:\:\:\:\:\:\:\:\:\:\:\:\:\:\:\:\:\:\:\:\:\:\:\:\:\:\:\:\:\:\:\:\:\:\:\:\:\:\:\:\:\:\:\:\:\:\:\:\:\:\:\:\:\:\:\:\:\:\:\:\:\:\:\:\:\:\:\:\:\:\:\:\:\:\:\:\:\:\:\:\:\:\:\:\:\:\:\:\:\:\:\:\:\:\:\:\:\:\:\:\:\:\:\:\:\:\:\:\:\:\:\:\:\:\:\:\:$$

then,

10 $$\:{{F}_{\text{r}}T}_{n}^{\propto\:}\left(t\right)=\:\sqrt{\frac{{c}_{n}}{w\left(t\right)}}{\stackrel{\sim}{{F}_{\text{r}}T}}_{n}^{\alpha\:}\left(t\right)$$

From Equ 10 into 9,

$$\:\sqrt{\frac{{c}_{n+1}}{w\left(t\right)}}{\stackrel{\sim}{{F}_{\text{r}}T}}_{n+1}^{\alpha\:}\left(t\right)=\left(2-4{t}^{\propto\:}\right)\sqrt{\frac{{c}_{n}}{w\left(t\right)}}{\stackrel{\sim}{{F}_{\text{r}}T}}_{n}^{\alpha\:}\left(t\right)-\sqrt{\frac{{c}_{n-1}}{w\left(t\right)}}{\stackrel{\sim}{{F}_{\text{r}}T}}_{n-1}^{\alpha\:}\left(t\right),$$

$$\:\sqrt{{c}_{n+1}}{\stackrel{\sim}{{F}_{\text{r}}T}}_{n+1}^{\alpha\:}\left(t\right)=\left(2-4{t}^{\propto\:}\right)\sqrt{{c}_{n}}{\stackrel{\sim}{{F}_{\text{r}}T}}_{n}^{\alpha\:}\left(t\right)-\sqrt{{c}_{n-1}}{\stackrel{\sim}{{F}_{\text{r}}T}}_{n-1}^{\alpha\:}\left(t\right)$$

where $$\:{c}_{n}=1\:for\:n\:\:1$$, then

11 $$\:{\stackrel{\sim}{{F}_{\text{r}}T}}_{n+1}^{\alpha\:}\left(t\right)=\left(2-4{t}^{\propto\:}\right){\stackrel{\sim}{{F}_{\text{r}}T}}_{n}^{\alpha\:}\left(t\right)-\sqrt{{c}_{n-1}}{\stackrel{\sim}{{F}_{\text{r}}T}}_{n-1}^{\alpha\:}\left(t\right)\:\:\:\:\:\:\:\:\:\:\:\:\:n=1,\:2,\:\dots\:,\:N-1$$

where $$\:{c}_{n-1}=2\:at\:n=1,\:and\:{c}_{n-1}=1\:for\:n\:\:2$$.

### Schwarz-Rutishauser method

The classical Gram-Schmidt method was modified to create the Schwarz-Rutishauser method^36,38^. Gram-Schmidt process is a method for converting a set of linearly independent vectors into an orthogonal or orthonormal set. This is extremely useful in many areas of mathematics and engineering, including solving linear systems, QR decomposition, and projection operations. It takes a matrix A as input, where each column represents a vector to be orthogonalized, and returns: Q - a matrix with orthonormal columns and R - an upper triangular matrix such that:

12 $$\:A\:=\:QR$$

where Q denotes an orthogonal matrix and R represents an upper triangular matrix. The orthogonal matrix Q is derived through a series of projection operations that can be expressed as:

13 $$\:{\text{q}}_{\text{i}}={\text{a}}_{\text{i}}-\sum\:={1}^{\text{i}-1}\text{\:proj\_q\:}\left({\text{a}}_{\text{i}}\right)$$

where $\:\text{proj\_q}\text{}\left({\text{a}}_{\text{i}}\right)$ represents the projection of vector a_i_ onto q and is defined as:

14 $$\:\text{proi\_q}\text{}\left({\text{a}}_{\text{i}}\right)={\text{a}}_{\text{i}},\text{q}/\text{q},\text{q}\cdot\:\text{q}={\text{a}}_{\text{i}},\text{q}/\Vert\:q{\Vert\:}^{2}\cdot\:\text{q}$$

The traditional Gram-Schmidt process suffers from two main limitations: it becomes numerically unstable and computationally intensive when processing large matrices. These challenges led researchers to develop the Schwarz-Rutishauser technique (also known as “Iterative Gram-Schmidt” or “Reorthogonalized Gram-Schmidt”), which was specifically designed to provide better numerical stability while reducing the computational burden typically associated with Gram-Schmidt orthogonalization methods.

The orthogonalized vectors in Q are computed by subtracting from each vector $a^{\rightarrow}_{i}$the sum of its projections onto previous orthogonal vectors. The normalized vector then becomes:

15 $$\:\widehat{{\text{q}}_{\text{i}}}={\text{q}}_{\text{i}}/\parallel{\text{q}}_{\text{i}}\parallel$$

While the classical Gram-Schmidt process provides a conceptual foundation for orthogonalization, it suffers from two critical limitations in practical applications: numerical instability when processing large matrices and computational inefficiency. The Schwarz-Rutishauser algorithm addresses these limitations by implementing a more numerically robust approach.

A key improvement in the Schwarz-Rutishauser method is the mathematical simplification achieved by eliminating the squared norm division from the projection components. This can be expressed as:

16 $$\:{\text{q}}_{\text{i}}={\text{a}}_{\text{i}}-\sum\:\:={1}^{\text{i}-1}{\text{r}}_{\text{i}}\cdot\:{\text{q}}^{\wedge\:}$$

Where the specific elements in the column vector $\:r$ can be identified through:

17 $$\:{r}_{i}={a}_{i},{\text{q}}^{\wedge\:}$$

Substituting this relationship into the previous equation yields:

18 $$\:{\text{q}}_{\text{i}}={\text{a}}_{\text{i}}-\sum\:\:={1}^{\text{i}-1}\:{\text{a}}_{\text{i}},{\text{q}}^{\wedge\:}\:\cdot\:{\text{q}}^{\wedge\:}$$

The computational advantage emerges from the fact that a_i_ initially equals q_i_, allowing for an efficient recursive computation of q_i_^k^ and r_i_ using the following relationships:

19 $$\begin{aligned}&{r}_{i}={q}_{i}^{k-1},{q}^{\wedge}\\&{q}_{i}^{k}={q}_{i}^{k-1}-{r}_{i}\cdot\:{q}^{\wedge}\end{aligned}$$

This recursive approach eliminates the need for summation in the original equation. The diagonal elements in R are computed as the norm of the corresponding orthogonalized vector:

20 $$\:{\text{r}}_{\text{i}\text{i}}=\parallel{\text{q}}_{\text{i}}\text{i}\parallel$$

The Schwarz-Rutishauser algorithm achieves superior numerical stability while maintaining computational efficiency compared to traditional QR decomposition methods. This makes it particularly well-suited for processing large-scale bio-signal data where both accuracy and processing speed are critical considerations.

## Proposed reconstruction and watermarking algorithm using fractional Tchebichef orthogonal moments and the Schwarz-Rutishauser method

### Proposed reconstruction algorithm

The proposed approach for signal reconstruction generally consists of two primary components: the Fractional Tchebichef orthogonal moments (FrTMs) and the Schwarz-Rutishauser method. FrTMs have been used for feature extraction from signals, whereas Schwarz-Rutishauser is employed to maintain stability with less complexity. The proposed reconstruction algorithm is delineated as consisting of five phases. The pseudo-code of the proposed algorithm is outlined below as Algorithm 1.

**Initially**, define the polynomial order’s upper bounds, represented by (N) and ($\:{\text{N}}_{\text{m}\text{a}\text{x}})$.

**Next**, compute two fundamental initial conditions: the fractional Tchebichef functions $\:{\stackrel{\sim}{{F}_{\text{r}}T}}_{0}^{\alpha\:}\left(t\right)$ and$\:\:{\stackrel{\sim}{{F}_{\text{r}}T}}_{1}^{\alpha\:}\left(t\right)$, using their respective mathematical formulations involving fractional powers and time variables.

$$\:{\stackrel{\sim}{{F}_{\text{r}}T}}_{0}^{\alpha\:}\left(t\right)=\frac{1}{\sqrt{2}}\:\frac{{t}^{\frac{\propto\:}{4}-\frac{1}{2}}}{1-{t}^{\propto\:}}\:\:\:\:\:\:\:\:\:\:\:\:$$

$$\:{\stackrel{\sim}{{F}_{\text{r}}T}}_{1}^{\alpha\:}\left(t\right)=\frac{{t}^{\frac{\propto\:}{4}-\frac{1}{2}}}{1-{t}^{\propto\:}}\:(1-2{t}^{\propto\:}).$$

**The third phase** involves deriving higher-order polynomials based on previously calculated terms. This recursive computation uses a relationship between consecutive terms and scaling coefficients.

$$\:{\stackrel{\sim}{FT}}_{n+1}^{\alpha\:}\left(t\right)=\left(2-4{t}^{\propto\:}\right){\stackrel{\sim}{FT}}_{n}^{\alpha\:}\left(t\right)-\sqrt{{c}_{n-1}}{\stackrel{\sim}{FT}}_{n-1}^{\alpha\:}\left(t\right)\:\:\:\:\:\:\:\:\:\:\:\:\:\:\:\:\:\:\:\:\:n=1,\:2,\:\dots\:,\:N-1.$$

**In the fourth step**, implement the Schwarz-Rutishauser algorithm to generate and normalize an orthogonal matrix Q. This process involves calculating inner products between vectors and performing sequential normalization operations.

$$\:{r}_{n,m}=\:\langle{\stackrel\rightharpoonup{Q}}_{1:L,n}^{T},{\stackrel\rightharpoonup{Q}}_{1:L,m}\rangle$$

$$\:{Q}_{1:L,m}={Q}_{1:L,m}-{r}_{n,m}\times\:{Q}_{1:L,n}$$

$$\:{Q}_{1:L,m}={Q}_{1:L,m}/ \parallel{Q}_{1:L,m}\parallel$$

**Finally**, the fractional Tchebichef moments are calculated by applying them to matrix Q, extracting signal features through a summation over the domain.

$$\:{\widehat{\:{\text{F}}_{\text{r}}\text{T}\text{M}}}_{n}=\:\sum_{x=0}^{N-1}\:{\stackrel{\sim}{{F}_{\text{r}}T}}_{n}^{\alpha\:}\left(t\right)\:s\left(x\right),\:\:\:\:\:\:\:\:\:\:n=\text{0,1},2,\dots\:,N-1.\:\:$$

To reconstruct the original signal$\:\widehat{\text{s}}\left(\text{x}\right)$, apply the inverse Fractional Tchebichef transform, which combines the computed moments with their corresponding polynomial terms.

$$\:\widehat{\text{s}}\left(\text{x}\right)=\:\sum_{n=0}^{{\text{n}}_{\text{m}\text{a}\text{x}}}{\widehat{\:{\text{F}}_{\text{r}}\text{T}\text{M}}}_{n}\:{\stackrel{\sim}{{F}_{\text{r}}T}}_{n}^{\alpha\:}\left(t\right)\:,\:\:\:\:\:\:\:\:\:\:x=\text{0,1},2,\dots\:,N-1.$$

### Proposed watermarking algorithm

**Fig. 1:**
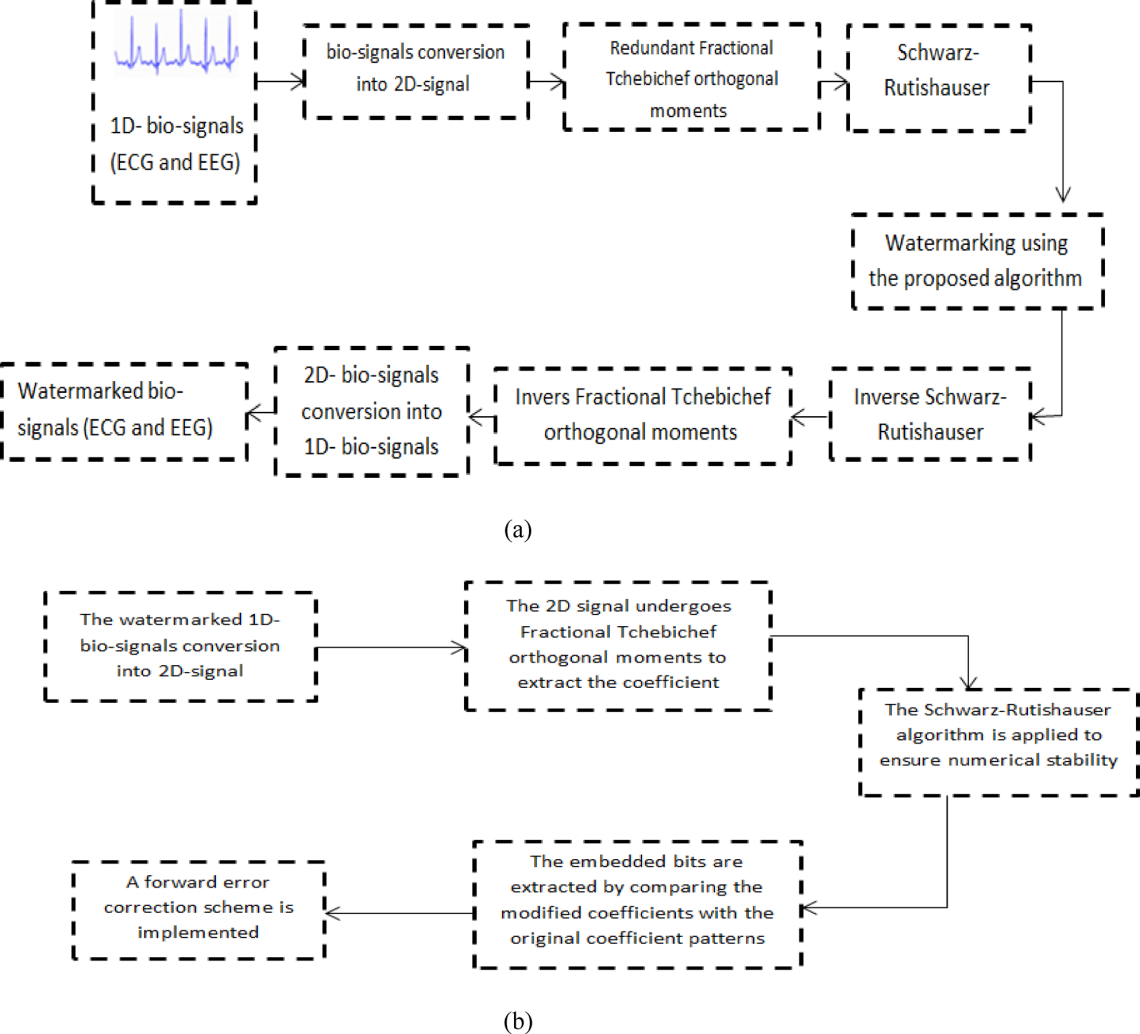
(**a**) Diagram of the watermarking embedding process (**b**) Diagram of the watermarking extraction process.

Figure 1 outlines the watermark integration process, (a) Diagram of the watermarking embedding process (b) Diagram of the watermarking extraction process. In the embedding process, the first step involves the bio-signals (i.e., ECG and EEG) conversion into 2D-signal. This process likely involves techniques such as Gramian Angular Field (GFA)^39^ or Recurrence Plots to transform the 1-dimensional ECG waveform into a 2-dimensional matrix representation. GFA approach have been used, it is a method that transforms time series data (like ECG or EGG signals) into a 2D image representation. In signal watermarking, the conversion of a 1D signal to 2D and back again (by using inverse transform) serves several important purposes, for example: Enhanced embedding capacity where, 2D representations provide more embedding space for watermark information. By distributing the watermark across both dimensions, you can embed more data while maintaining imperceptibility. This 2D transformation can capture important temporal and amplitude patterns in the bio-signals that may be difficult to discern in the original 1D format. The next step applies “Redundant Fractional Tchebichef orthogonal moments” to the 2D signal. Orthogonal moments are mathematical functions that extract features and represent the signal more compactly. The “Redundant Fractional” aspect likely refers to using oversampled or redundant moment calculations to improve robustness to noise and distortions. The figure indicates “Watermarking is using the proposed algorithm” after the feature extraction. This suggests applying a digital watermarking technique in which additional information is imperceptibly embedded into the bio-signals. This could enable applications such as authentication, ownership verification, or secure transmission of the ECG data. Finally, the “Inverse Schwarz-Rutishauser” step is a reconstruction process that converts the modified 2D representation back into a 1D bio-signals format, potentially for further clinical analysis or storage.

**Fig. 2:**
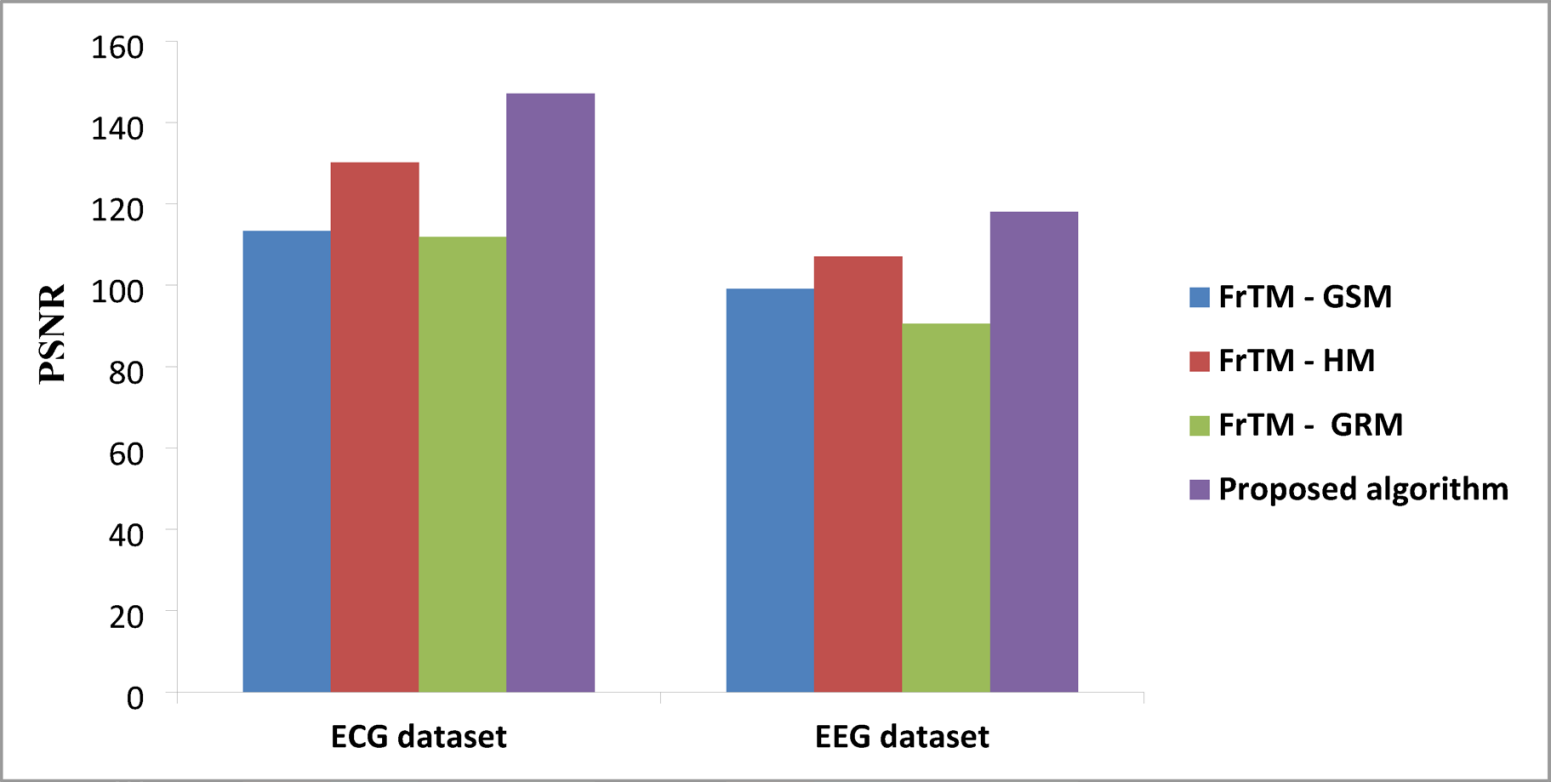
The average values of the performance metric (PSNR) for the proposed algorithm against the three traditional approaches: FrTMs-GSM, FrTMs-HM, and FrTMs-GRM.

The extraction of the embedded watermark follows a reverse process of the embedding algorithm, as illustrated in Fig. 2 below. The extraction process consists of the following steps. The watermarked 1D ECG/EEG signal is first transformed into a 2D representation using the same conversion technique employed during embedding. The 2D signal undergoes FrTM transformation to extract the coefficient domain where the watermark was embedded. The Schwarz-Rutishauser algorithm is applied to ensure numerical stability during the extraction process. The embedded bits are extracted by comparing the modified coefficients with the original coefficient. A forward error correction scheme is implemented to handle potential bit errors during extraction, improving the robustness against noise and signal processing operations. The extraction process is designed to be blind, meaning the original signal is not required for watermark extraction, which makes it particularly suitable for telemonitoring applications in healthcare systems.

The proposed watermarking algorithm utilizes a binary watermark sequence as the embedded information. This watermark can represent ownership identification data, patient information, or authenticity verification codes. The algorithm supports watermark embedding with a capacity of up to 128 bits for ECG signals and 256 bits for EEG signals, depending on the signal length and required imperceptibility level. The embedding capacity C of the watermark is determined based on the relationship between the signal length N and the embedding strength parameter α: C = ⌊N/α⌋.

where α is adaptively calculated based on the signal characteristics to maintain an optimal balance between imperceptibility and robustness. For the MIT-BIH arrhythmia dataset used in our experiments, we achieved an average embedding capacity of 0.025 bits per sample, which is sufficient for embedding essential patientinformation while maintaining high signal fidelity.

**Table:** 

**Algorithm 1**: The pseudo-code of the proposed reconstruction algorithm
1. Input the original signal$\:\:\mathbf{s}\left(\mathbf{x}\right)$. 2. Put the maximum value (*N*) of variable x. 3. Put a polynomial’s order ($\:{\varvec{N}}_{\mathbf{m}\mathbf{a}\mathbf{x}}$). 4. For x←0 to *N*−1 do 5. Calculate $\:{\stackrel{\sim}{{\varvec{F}}_{\mathbf{r}}\varvec{T}}}_{0}^{\varvec{\alpha\:}}\left(\varvec{t}\right)$ using Equ.7. 6. Calculate $\:{\stackrel{\sim}{{\varvec{F}}_{\mathbf{r}}\varvec{T}}}_{1}^{\varvec{\alpha\:}}\left(\varvec{t}\right)$ using Equ.8. 7. For $\:\varvec{i}\:\leftarrow\:2$ to $\:{\varvec{N}}_{\mathbf{m}\mathbf{a}\mathbf{x}}-1$ do 8. Calculate $\:{\stackrel{\sim}{{\varvec{F}}_{\mathbf{r}}\varvec{T}}}_{\varvec{n}}^{\varvec{\alpha\:}}\left(\varvec{t}\right)$ using Equ.11. 9. End For 10. $\:\varvec{F}\varvec{T}={\stackrel{\sim}{{\varvec{F}}_{\mathbf{r}}\varvec{T}}}_{\varvec{n}}^{\varvec{\alpha\:}}\left(\varvec{t}\right)$ 11. For *i*$\:\:\leftarrow\:1\:$to N do 12. $\:{\:\varvec{Q}}_{1:\varvec{N},\varvec{i}}={\varvec{F}\varvec{T}}_{1:\varvec{N},\varvec{i}}$ 13. For $\:\varvec{j}\leftarrow\:0$ to $\:\varvec{i}-1$ do 14. $\:{\varvec{r}}_{\varvec{i},\varvec{j}}=\:\langle{\stackrel{\rightharpoonup}{\varvec{Q}}}_{1:\varvec{N},\varvec{i}}^{\varvec{T}},{\stackrel{\rightharpoonup}{\varvec{Q}}}_{1:\varvec{N},\varvec{j}}\rangle$ 15. $\:{\varvec{Q}}_{1:\varvec{N},\varvec{j}}={\varvec{Q}}_{1:\varvec{N},\varvec{j}}-{\varvec{r}}_{\varvec{i},\varvec{j}}\times\:{\varvec{Q}}_{1:\varvec{N},\varvec{i}}$ 16. End For 17. $\:{\varvec{Q}}_{1:\varvec{N},\varvec{j}}={\varvec{Q}}_{1:\varvec{N},\varvec{j}}/\parallel{\varvec{Q}}_{1:\varvec{N},\varvec{j}}\parallel$ 18. End For 19. End For 20. Apply Fractional Tchebichef moments $\:{\widehat{\:{\mathbf{F}}_{\mathbf{r}}\mathbf{T}\mathbf{M}}}_{\varvec{n}}$ using Equ.1. 21. Apply the inverse of Fractional Tchebichef moments to obtain $\:\widehat{\varvec{s}}\left(\varvec{x}\right)\:$by Equ.2.

## Experimental results and discussion

### Datasets

The experimental validation of the proposed algorithm utilized two established benchmark datasets from the field of medical signal processing: the MIT-BIH Arrhythmia Database^40^ and the CHB-MIT Scalp EEG Database^41^.

- The MIT-BIH Arrhythmia Database consists of 48 half-hour excerpts of two-channel ambulatory ECG recordings. The recordings were digitized at 360 samples per second per channel with 11-bit resolution over a 10 mV range. For our experimental evaluation, we selected seven representative recordings (Rec. 100, 104, 109, 115, 124, 202, and 230) that encompass a diverse range of cardiac conditions and signal morphologies. These particular records were chosen because they contain various arrhythmias, different QRS morphologies, and varying signal-to-noise ratios, thereby providing a comprehensive tested for our reconstruction and watermarking algorithms.
- The CHB-MIT Scalp EEG Database consists of EEG recordings from pediatric subjects with intractable seizures monitored at Boston Children’s Hospital. The recordings were collected from 23 patients (5 males, ages 3–22; and 18 females, ages 1.5–19) during several days of continuous monitoring. The EEG signals were sampled at 256 Hz with 16-bit resolution using the International 10–20 system of electrode placement.

### Performance evaluation metrics

In the experiments, the Mean Square Error (MSE), Kullback–Leibler (KLD), Jensen-Shannon distances (JSD), Peak Signal-to-Noise Ratio (PSNR), Bit error rate (BER), and Percentage Residual Difference (PRD) were used to evaluate the efficiency of the proposed algorithm^42,43^.

- MSE :

21 $$\:\text{M}\text{S}\text{E}=\frac{1}{\text{N}}\sum\:_{\text{x}=0}^{\text{N}-1}\:\left(\text{s}\right(\text{x})-\text{s}\\hat :(\text{x}){)}^{2}$$

- PSNR :

22 $$\:\text{P}\text{S}\text{N}\text{R}=20\times\:{\text{log}}_{10}\frac{max\left|s\left(x\right)\right|}{\sqrt{MSE}}$$

- DKL :

23 $$\:{D}_{\text{K}\text{L}}\left(s\right(x)\Vert\:\text{s}\\hat :(\text{x}\left)\right)={\int\:}_{-{\infty\:}}^{+{\infty\:}}\:s\left(x\right)\times\:\text{l}\text{o}\text{g}\frac{s\left(x\right)}{s\\hat :\left(x\right)}\text{d}y$$

- JSB :

24 $$\:JSD\left(s\right(x)\parallel \hat {s} (x\left)\right)=\:\frac{1}{2}\int\:s\left(x\right)\text{l}\text{o}\text{g}\left(\frac{s\left(x\right)}{\frac{1}{2}\left(s\right(x)+ \hat {s} (x\left)\right)}\right)\text{d}x+\frac{1}{2}\int\: \hat {s} \left(x\right)\text{l}\text{o}\text{g}\left(\frac{\hat {s} \left(x\right)}{\frac{1}{2}\left(s\right(x)+\hat {s} (x\left)\right)}\right)\text{d}x$$

where $\:s\left(x\right)\:$and $$\:s\\hat :\left(x\right)$$ the original and reconstructed signal, respectively.

- BER:

25 $$\:\text{B}\text{E}\text{R}=\frac{{\sum\:}_{i,j}\left(I\left(i,j\right)\text{o}J\right(i,j\left)\right)}{M*N}$$

The bit error rate (BER) quantifies the frequency of errors occurring when digital data are received during transmission. This metric evaluates how many bits are incorrectly received compared to the original signal, considering factors like signal degradation and interference. The BER calculation compares two signals, typically labeled *I* and *J*, with dimensions i and j, respectively, to determine the proportion of transmission errors.

- PRD:

26 $$\:\text{P}\text{R}\text{D}=\sqrt{\frac{\sum\:i,j{\left|I\left(i,j\right)-J(i,j)\right|}^{2}}{\sum\:i,j\sum\:_{i,j}{I\left(i,j\right)}^{{2}^{2}}}\:\:}\times\:100$$

The Percentage Residual Difference (PRD) measures how much a watermarked ECG signal deviates from its original form. This metric is calculated by examining the squared differences between the initial ECG signals *I* and its watermarked version *J*.

### Results of the proposed algorithm compared to FrCM with QR decomposition methods

**Fig. 3:**
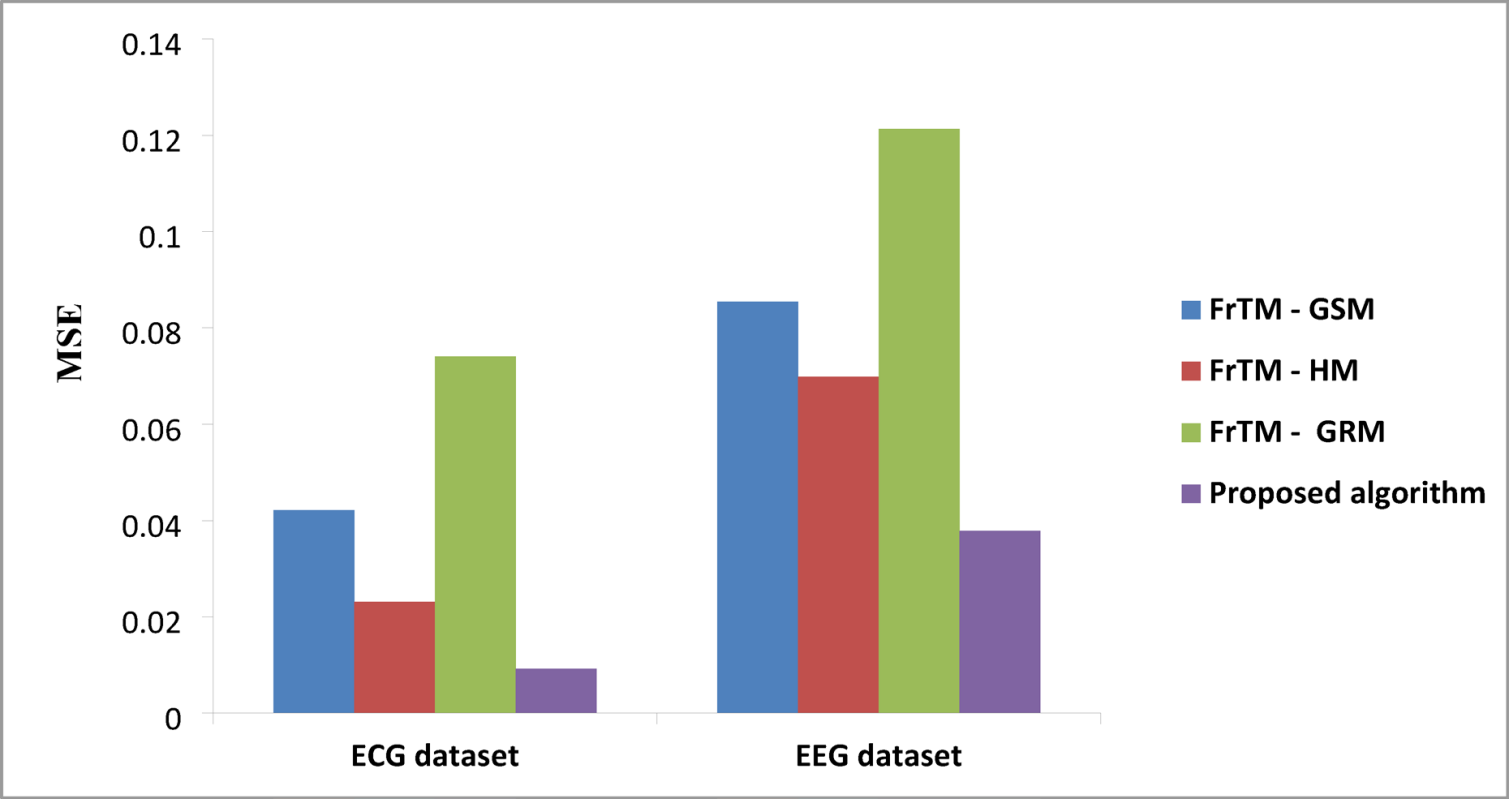
The average values of the performance metric (MSE) for the proposed algorithm against the three traditional approaches: FrTMs-GSM, FrTMs-HM, and FrTMs-GRM.

This section performs an experimental investigation of the proposed algorithm to evaluate its performance. The comparative results are compared to Fractional order Discrete Tchebichef moments with QR decomposition (FrCM – Gram-Schmidt Method (GSM), FrCM – Householder Method (HM), and FrCM - Given Rotations Method(GRM)). A comparative analysis of the performance metrics is documented across two key datasets. When evaluating the MIT-BIH Arrhythmia signals, our methodology outperformed traditional FrTMs approaches, as evidenced in Table 1. Similarly, the new approach demonstrated superior results when tested against FrCM implementations using GSM, HM, and GRM techniques on the CHB-MIT Scalp dataset (Table 2). Specifically, the quantitative assessment revealed peak performance with a PSNR of 147.0814 and MSE of 0.0092 for the first dataset while achieving a PSNR of 118.449 and MSE of 0.0379 in the second dataset. The comparative visualization of average PSNR and MSE metrics across all methods is illustrated in Figs. 2 and 3, respectively. These visual representations further confirm our algorithm’s enhanced signal reconstruction quality across both datasets, surpassing the performance of alternative FrCM implementations utilizing GSM, HM, and GRM approaches.

**Table 1:** Comparative analysis of the proposed algorithm versus FrTMs with (GSM, HM, and GRM) on the MIT-BIH arrhythmia dataset.

Signal	FrTM - GSM		FrTM - HM		FrTM - GRM		Proposed algorithm	
	MSE	PSNR	MSE	PSNR	MSE	PSNR	MSE	PSNR
Rec. 100	0.0436	140.587	0.0257	152.92	0.0554	120.382	0.0189	163.71
Rec. 104	0.1072	97.0226	0.0371	119.391	0.2807	117.884	0.0245	114.92
Rec. 109	0.0356	125. 981	0.0157	138.722	0.0380	113.35	0.0016	145.46
Rec. 115	0.0407	109.967	0.0191	110.783	0.0441	98.196	0.0096	148.82
Rec. 124	0.0182	115.814	0.0296	128.811	0.0301	111.169	0.0041	156.2
Rec. 202	0.0199	113.702	0.0175	126.587	0.0414	121.452	0.0025	151.41
Rec. 230	0.0237	114.741	0.0171	133.603	0.0286	100.872	0.0033	149.05
Average	**0.04127**	**113.556**	**0.0231**	**130.116**	**0.0740**	**111.9007**	**0.0092**	**147.0814**

**Table 2:** Comparative analysis of the proposed algorithm versus FrTMs with (GSM, HM, and GRM) on the CHB-MIT dataset.

Signal	FrTM - GSM		FrTM - HM		FrTM - GRM		Proposed algorithm	
	MSE	PSNR	MSE	PSNR	MSE	PSNR	MSE	PSNR
Chb01-01	0.1747	107.587	0.1062	116.973	0.1998	101.587	0.0862	138.185
Chb01-02	0.0671	81.0226	0.0571	89.751	0.0984	74.0226	0.0338	111.9961
Chb03-34	0.1183	101. 981	0.1007	112. 856	0.1806	101. 981	0.0.711	127.653
Chb10-02	0.0223	98.967	0.0298	106.997	0.0654	91.967	0.0154	103.722
Chb10-12	0.0401	103.814	0.0422	115.936	0.0801	92.814	0.0251	114.643
Chb20-22	0.1609	97.702	0.1374	108.881	0.1847	87.702	0.1006	116.947
Chb24-07	0.0144	95.741	0.0155	103.914	0.0401	89.741	0.0105	115.997
Average	**0.0854**	**99.056**	**0.0698**	**107.0753**	**0.1213**	**90.556**	**0.0379**	**118.449**

Table 3 presents the KLD and JSD values obtained for the MIT-BIH Arrhythmia dataset signals, comparing our proposed algorithm with existing FrTM implementations using GSM, HM, and GRM approaches. Similarly, Table 4 presents the KLD and JSD values for the CHB-MIT Scalp EEG dataset. The information-theoretic analysis provides additional evidence supporting the superiority of our proposed algorithm. For the MIT-BIH Arrhythmia dataset, our method achieves an average KLD of 0.0199 and JSD of 0.0195, representing improvements of 58.1% and 57.2% respectively compared to the next best method (FrTM-HM with KLD = 0.0475 and JSD = 0.0456). Similarly, for the CHB-MIT Scalp EEG dataset, our algorithm exhibits an average KLD of 0.0979 and JSD of 0.0591, representing improvements of 29.9% and 23.1% respectively compared to FrTM-HM. These results indicate that our proposed method preserves the statistical properties of the original signals significantly better than existing approaches. The lower KLD and JSD values confirm that the reconstructed signals not only minimize error in terms of amplitude differences (as measured by MSE and PSNR) but also maintain the underlying probability distributions of the original signals. This property is particularly important for biomedical signals where preserving statistical characteristics is crucial for accurate clinical interpretation and automated analysis. The superior performance in information-theoretic metrics can be attributed to two key factors in our method: the improved numerical stability provided by the normalized fractional-order Tchebichef moments, which reduces distortions in the moment space; and the enhanced orthogonality maintenance achieved through the Schwarz-Rutishauser method, which ensures that the statistical properties of the signals are accurately represented in the transform domain. These findings further validate the effectiveness of our approach for biomedical signal processing applications where maintaining the statistical integrity of the signals is paramount for accurate diagnosis and analysis.

**Table 3:** Comparative analysis of the proposed algorithm versus FrTMs with (GSM, HM, and GRM) on the MIT-BIH arrhythmia in KLD and JSD.

Signal	FrTM - GSM		FrTM - HM		FrTM - GRM		Proposed algorithm	
	KLD	JSD	KLD	JSD	KLD	JSD	KLD	JSD
Rec. 100	0.0872	0.0623	0.0531	0.0482	0.1103	0.0742	0.0392	0.0310
Rec. 104	0.2144	0.1073	0.0785	0.0613	0.5614	0.1712	0.0519	0.0427
Rec. 109	0.0712	0.0584	0.0327	0.0381	0.0760	0.0592	0.0051	0.0093
Rec. 115	0.0814	0.0637	0.0398	0.0417	0.0882	0.0682	0.0210	0.0214
Rec. 124	0.0364	0.0413	0.0592	0.0518	0.0602	0.0581	0.0087	0.0126
Rec. 202	0.0398	0.0429	0.0350	0.0396	0.0828	0.0648	0.0053	0.0086
Rec. 230	0.0474	0.0462	0.0342	0.0387	0.0572	0.0528	0.0078	0.0112
Average	**0.0825**	**0.0603**	**0.0475**	**0.0456**	**0.1480**	**0.0784**	**0.0199**	**0.0195**

**Table 4:** Comparative analysis of the proposed algorithm versus FrTMs with (GSM, HM, and GRM) on the CHB-MIT dataset in KLD and JSD.

Signal	FrTM - GSM		FrTM - HM		FrTM - GRM		Proposed algorithm	
	KLD	JSD	KLD	JSD	KLD	JSD	KLD	JSD
Chb01-01	0.3494	0.1279	0.2124	0.0985	0.3996	0.1384	0.1724	0.0849
Chb01-02	0.1342	0.0817	0.1142	0.0751	0.1968	0.0912	0.0676	0.0527
Chb03-34	0.2366	0.1086	0.2014	0.0978	0.3612	0.1302	0.1422	0.0791
Chb10-02	0.0446	0.0462	0.0596	0.0531	0.1308	0.0792	0.0308	0.0342
Chb10-12	0.0802	0.0629	0.0844	0.0647	0.1602	0.0882	0.0502	0.0431
Chb20-22	0.3218	0.1208	0.2748	0.1093	0.3694	0.1319	0.2012	0.0918
Chb24-07	0.0288	0.0382	0.0310	0.0396	0.0802	0.0627	0.0210	0.0281
Average	0.1708	0.0838	0.1397	0.0769	0.2426	0.1031	0.0979	0.0591

### Results of the proposed algorithm compared to FrCM with QR decomposition methods at different orders

To investigate the proposed algorithm’s superior reconstruction quality further, we compared it with other methods that use QR decomposition (GSM, HM, and GRM) in reconstruction at different orders. We use the “Rec. 100” signal as an example from the MIT-BIH Arrhythmia dataset and the “Chb01-01” signal as an example from the CHB-MIT Scalp dataset.

**Fig. 4:**
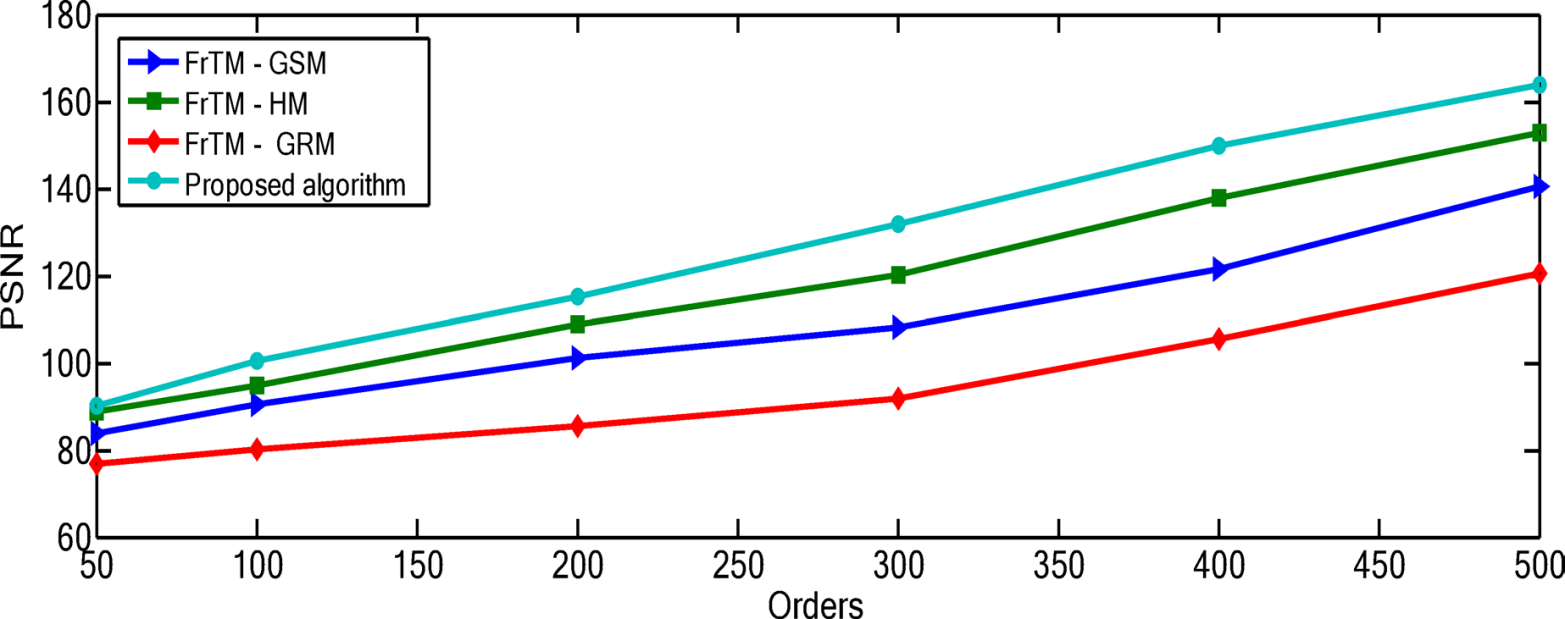
PSNR values of the reconstructed signal “Rec. 100” for the proposed algorithm against the three traditional approaches: FrTMs-GSM, FrTMs-HM, and FrTMs-GRM at various orders.

**Fig. 5:**
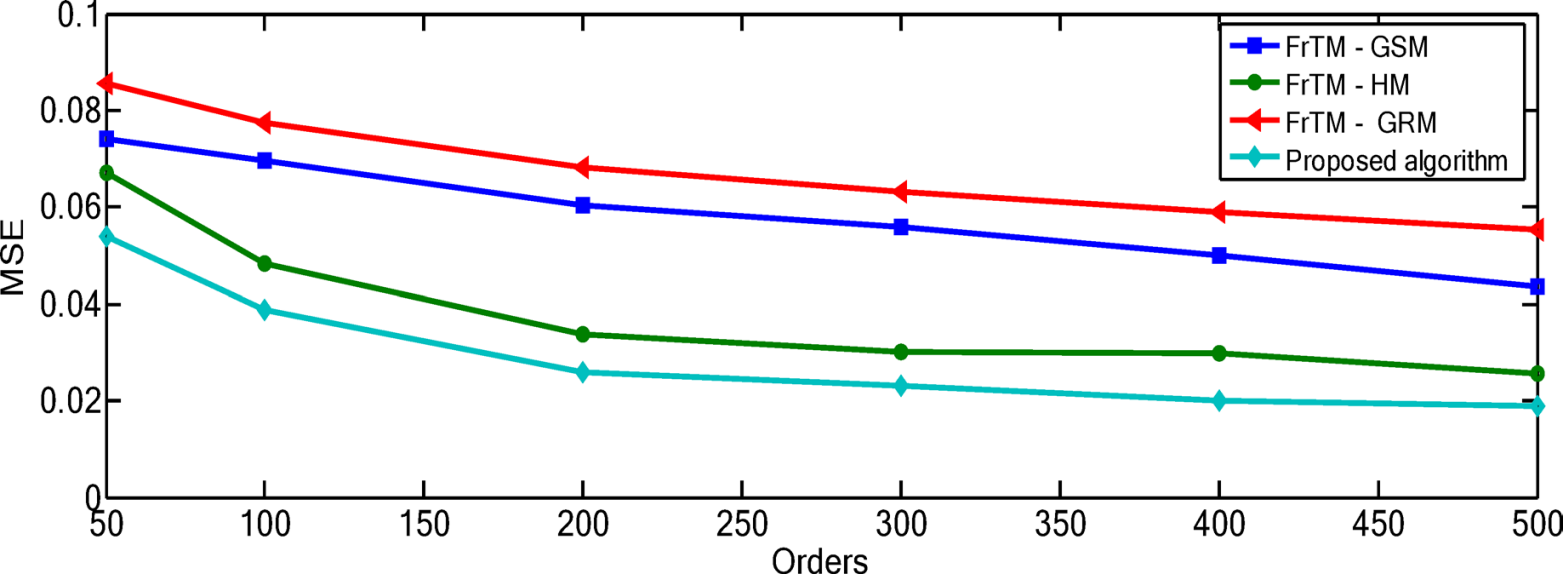
MSE values of the reconstructed signal"Rec. 100” for the proposed algorithm against the three traditional approaches: FrTMs-GSM, FrTMs-HM, and FrTMs-GRM at various orders.

**Fig. 6:**
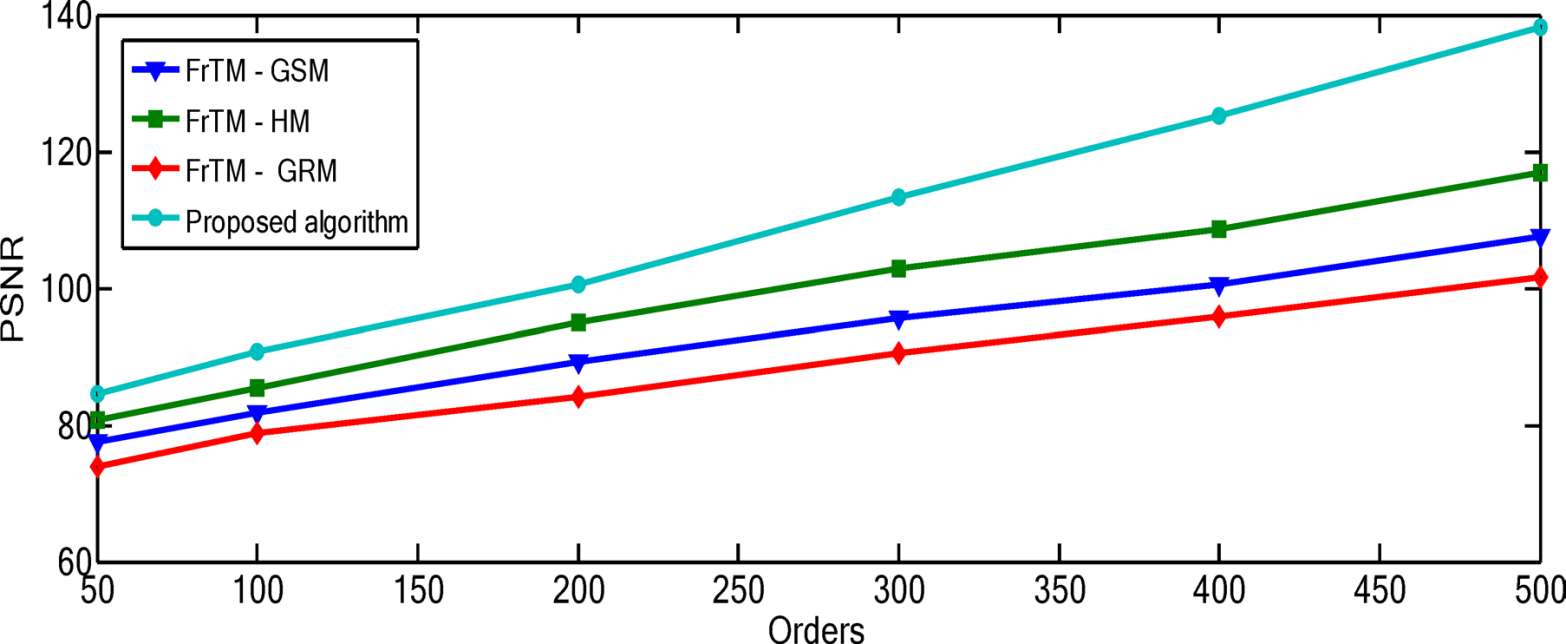
PSNR values of the reconstructed signal"Chb01-01” for the proposed algorithmagainst the three traditional approaches: FrTMs-GSM, FrTMs-HM, and FrTMs-GRM at various orders.

**Fig. 7:**
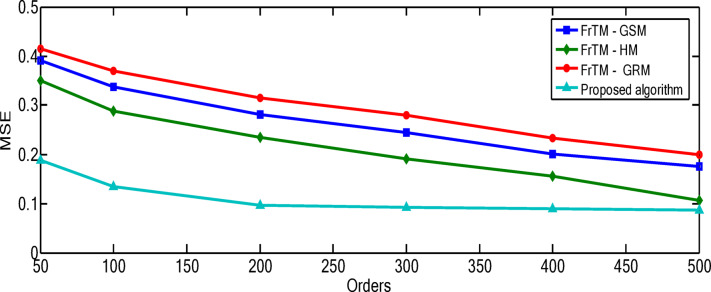
MSE values of the reconstructed signal “Chb01-01” for the proposed algorithm against the three traditional approaches: FrTMs-GSM, FrTMs-HM, and FrTMs-GRM at various orders.

The relationship between polynomial order and reconstruction quality shows a consistent pattern of improvement, but with diminishing returns at higher orders. For the “Rec. 100” signal, the proposed algorithm demonstrates particularly significant advantages at higher orders, with the performance gap widening most dramatically between orders 300–500. At order 500, the proposed algorithm achieves a PSNR of 163.71 compared to the next best competitor (FrTM-HM) at 152.92, representing a substantial 7.1% improvement. The rate of improvement across orders is non-linear for all methods. For example, with the proposed algorithm on the “Rec. 100” signal, the PSNR improvement from order 50 to 100 is approximately 10.3 dB, while from order 400 to 500 it’s approximately 13.9 dB. This accelerating improvement at higher orders suggests that the proposed method’s numerical stability advantages become more pronounced as complexity increases. The numerical results for the “Rec. 100” signal are depicted through Figs. 4 and 5. For the “Chb01-01” EEG signal, the performance patterns differ from the ECG signals, with all methods showing more gradual improvement as orders increase. This highlights how signal characteristics influence reconstruction performance, with the more complex and variable EEG signals presenting greater challenges to all methods, though the proposed algorithm maintains its advantage. The numerical results for the “Rec. 100” signal are depicted through Figs. 6 and 7. The MSE results reveal that error reduction follows different patterns for different methods. The proposed algorithm shows more consistent error reduction across the entire range of orders, while competing methods show plateauing effects at higher orders, suggesting accumulating numerical errors that limit their effectiveness. The computational efficiency advantage of the proposed algorithm becomes more significant at higher orders. This is particularly important for real-time applications, as the performance benefits come without the typical computational penalty associated with higher-order polynomials in traditional methods. These observations reinforce that the proposed algorithm’s combination of normalized Fractional Tchebichef Moments with the Schwarz-Rutishauser method provides both better reconstruction quality and greater numerical stability, with the advantages becoming more pronounced at higher polynomial orders and for more complex signals.

### Performance comparison of the proposed algorithm in reconstruction compared to existing algorithms

**Fig. 8:**
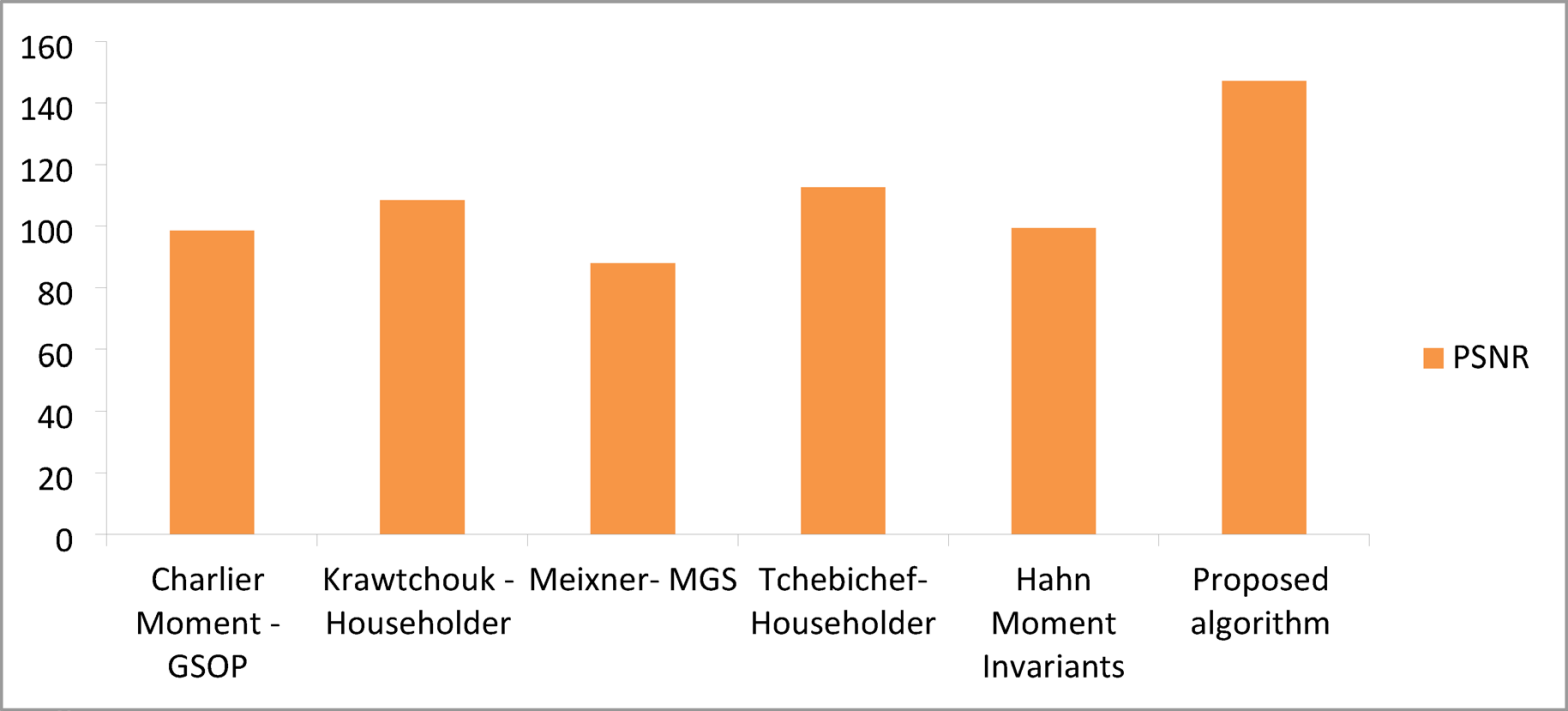
Comparison of PSNR average values of the proposed algorithm and other existing techniques.

**Fig. 9:**
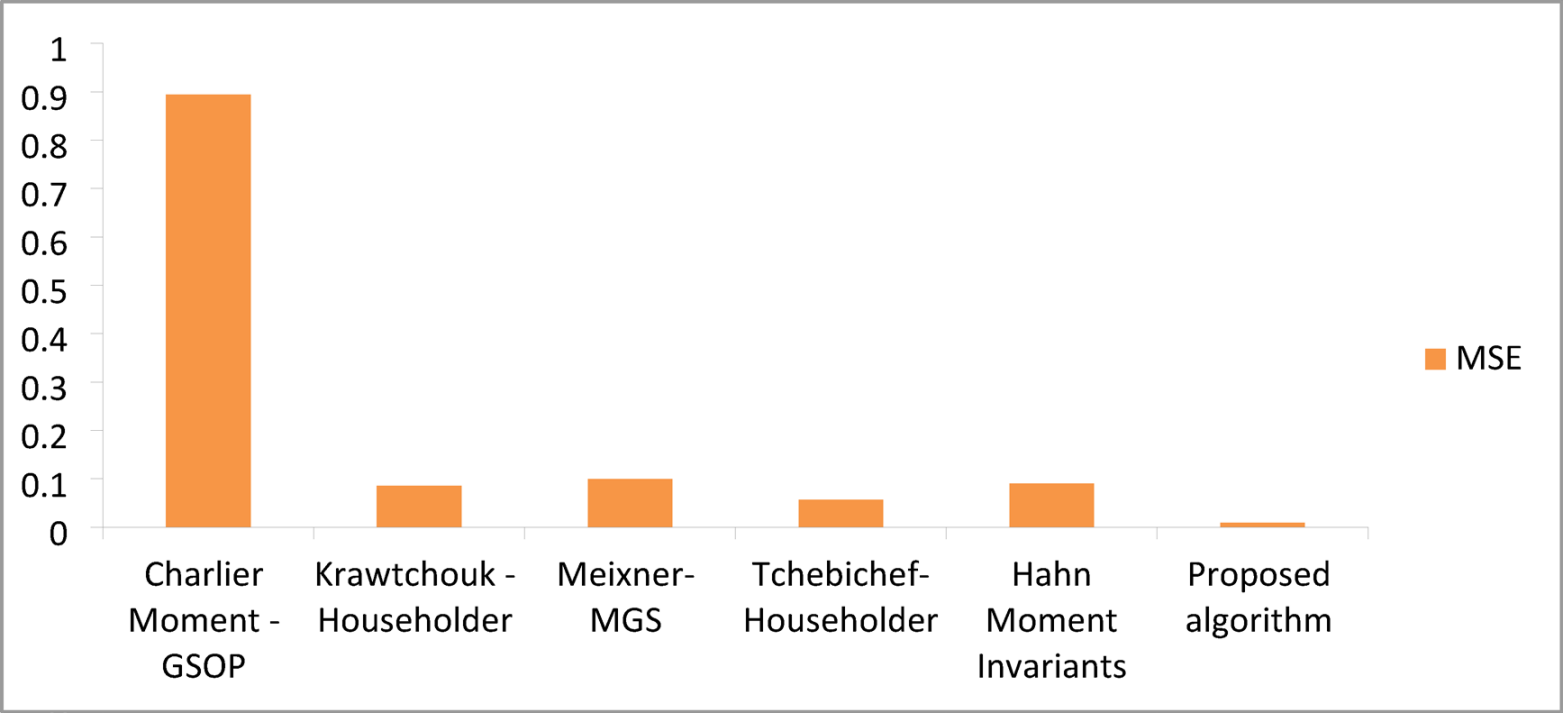
Comparison of MSE average values of the proposed algorithm and other existing techniques.

In this section, comparative results are provided to validate the superiority of the proposed algorithm in terms of reconstruction quality compared with other existing algorithms. Table 5 summarizes the comparative results with other available algorithms^4,22,43,44^ regarding reconstruction quality. A visual analysis of the comparative performance metrics is presented in Figs. 8 and 9, illustrating the PSNR and MSE measurements across different algorithmic approaches. The quantitative data in Table 5, supported by the graphical representations in Figs. 8 and 9, demonstrate the superior performance of our methodology. The enhanced reconstruction quality is evidenced by the ability of the algorithm to achieve exceptionally high PSNR values while maintaining minimal MSE measurements, indicating optimal signal reconstruction fidelity.

**Table 5:** Results of the introduced algorithm compared to FrTMs- GSM, FrTMs – HM, and FrTMs – GRM.in various orders.

Signal	Order	FrCM - GSM		FrCM - HM		FrCM - GRM		Proposed algorithm	
		**MSE**	**PSNR**	**MSE**	**PSNR**	**MSE**	**PSNR**	**MSE**	PSNR
**Rec. 100**	**50**	0.0742	83.906	0.0671	88.707	0.0855	76.996	0.0540	90.275
	**100**	0.0696	90.588	0.0483	94.809	0.0774	80.223	0.0389	100.603
	**200**	0.0604	101.119	0.0337	108.847	0.0681	85.661	0.0258	115.322
	**300**	0.0558	108.308	0.0301	120.014	0.0633	91.847	0.0231	131.874
	**400**	0.0501	121.339	0.0297	137.801	0.0591	105.658	0.0199	149.745
	**500**	0.0436	140.587	0.0257	152.92	0.0554	120.382	0.0189	163.71
**Chb01-01**	**50**	0.3914	77.548	0.3495	80.707	0.4147	73.971	0.1885	84.562
	**100**	0.3368	81.784	0.2887	85.322	0.3697	78.844	0.1341	90.743
	**200**	0.2811	89.339	0.2341	94.941	0.3141	84.117	0.0963	100.609
	**300**	0.2447	95.711	0.1914	102.801	0.2793	90.632	0.0921	113.401
	**400**	0.2011	100.558	0.1551	108.7171	0.2337	95.811	0.0899	125.113
	**500**	0.1747	107.587	0.1062	116.973	0.1998	101.587	0.0862	138.185

### Performance comparison of the proposed watermarking algorithm compared to previous existing algorithms

Table 6 comprehensively compares watermarking algorithms using three key performance metrics (PRD, PSNR, and BER). The results demonstrate the algorithm’s superior performance regarding PSNR, (BER), and PRD. The proposed algorithm achieved a PSNR value of 66.847 dB, representing a notable improvement over existing methods. The substantial improvement over Khaldi et al. (48.380) and Natgunanathan et al. (49.81 dB) indicates that our algorithm maintains significantly better signal quality and introduces less distortion in the watermarked content. Regarding Bit Error Rate, the proposed algorithm demonstrates superior performance with a BER of 0.25, showing substantial improvement over competing methods. This represents a significant advancement compared to Mathivanan et al. (0.4522), Khaldi et al. (0.7812), and particularly Natgunanathan et al. (1.57). The lower BER indicates that the proposed algorithm achieves more reliable watermark extraction and better robustness against potential distortions or attacks. The PRD metric, which measures the deviation from the original signal, demonstrates that the proposed algorithm has a value of 0.301. While this is slightly higher than Mathivanan et al. (0.029), it represents a better balance when considered alongside the other performance metrics. The algorithm shows improved performance compared to Khaldi et al. (0.563) and maintains competitive performance with different methods while achieving superior results in PSNR and BER.

**Table 6:** Comparing the proposed algorithm with existing techniques in reconstruction.

Techniques	MSE	PSNR
**Hahn Moment Invariants (HMIs)** ^44^	0.0901	99.337
**Tchebichef - Householder ** ^22^	0.0568	112.641
**Charlier Moment - GSOP ** ^4^	0.894	98.511
**Krawtchouk - Householder ** ^22^	0.0851	108.376
**Meixner- MGS ** ^45^	0.0996	87.984
**Proposed algorithm**	0.0092	147.0814

**Fig. 10:**
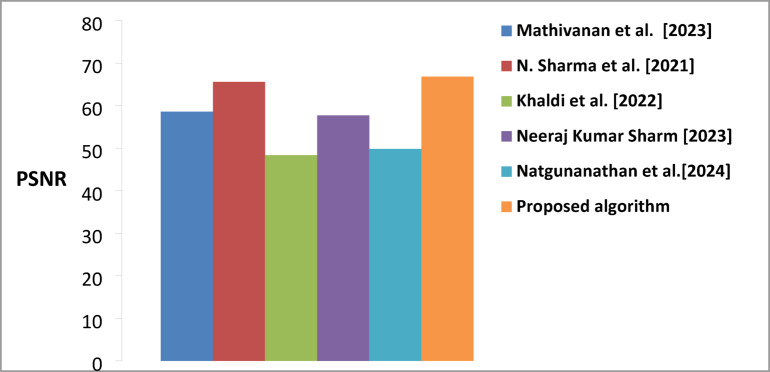
A comparison of PSNR average values was conducted between the proposed algorithm and existing Techniques.

**Fig. 11:**
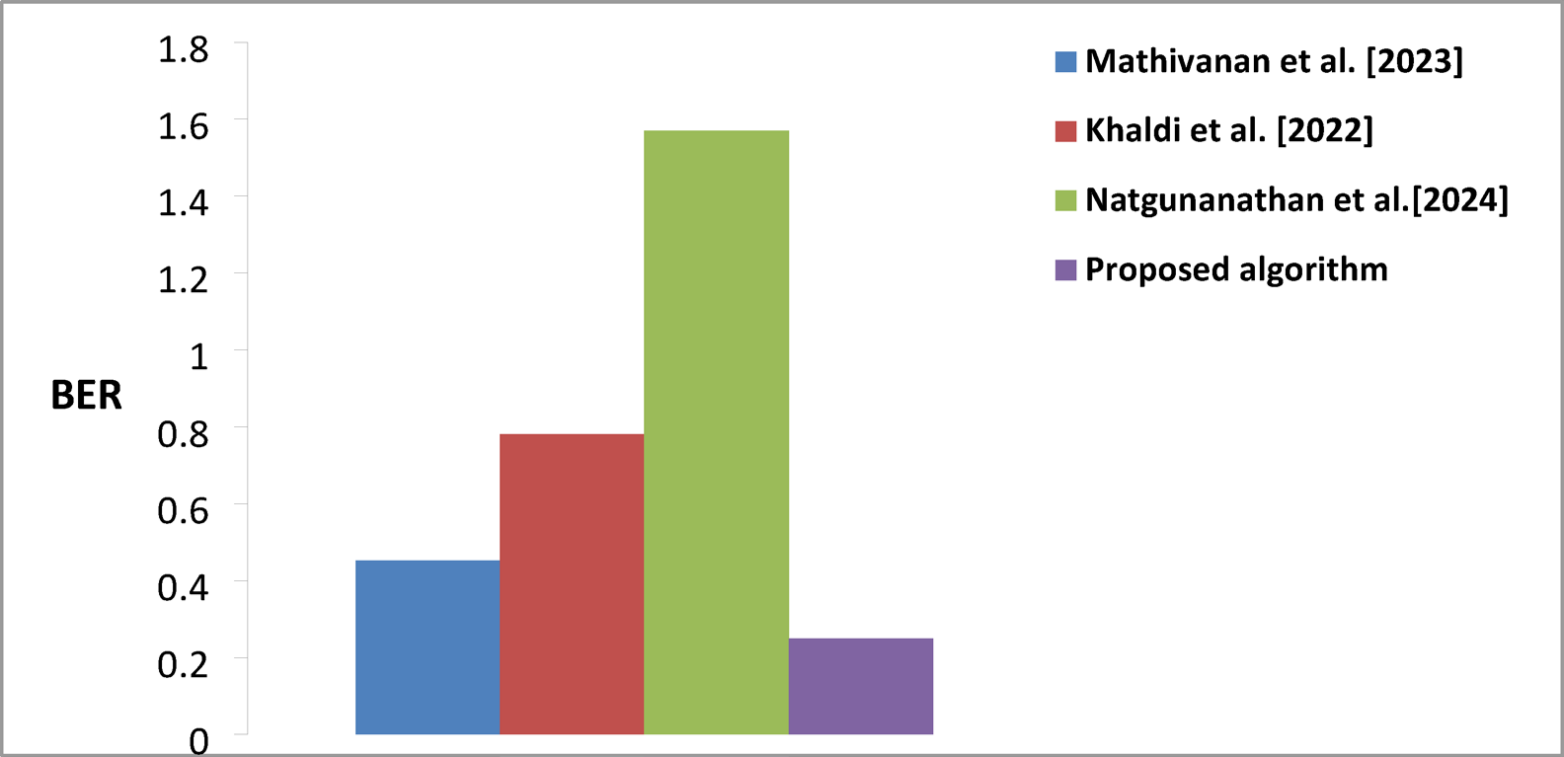
A comparison of BER average values was conducted between the proposed algorithm and existing Techniques.

**Fig. 12:**
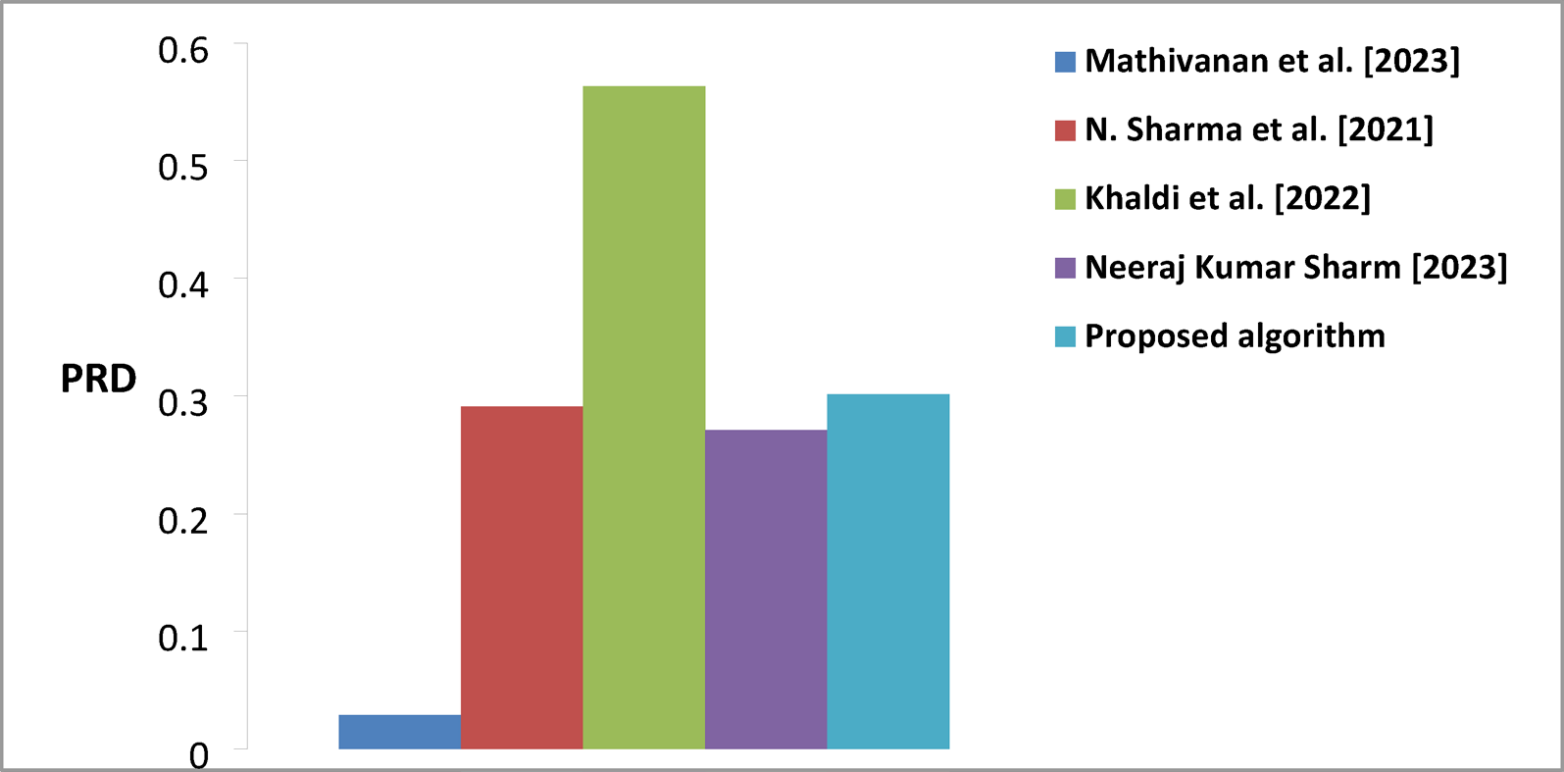
A comparison of PRD average values was conducted between the proposed algorithm and existing Techniques.

Figure 10 show demonstrates the evolution of PSNR performance in watermarking algorithms. The bar graph clearly illustrates the superiority of the proposed algorithm in terms of PSNR. Figure 11 illustrates the visual representation of BER for the proposed and existing methods. The proposed algorithm shows notably lower BER than others, significantly improving watermark extraction reliability. The variation in signal distortion across different algorithms is illustrated in Fig. 12. Mathivanan et al. show exceptionally low PRD, but the proposed algorithm maintains competitive PRD while excelling in other metrics.

### Results of computational efficiency for the proposed algorithm

**Fig. 13:**
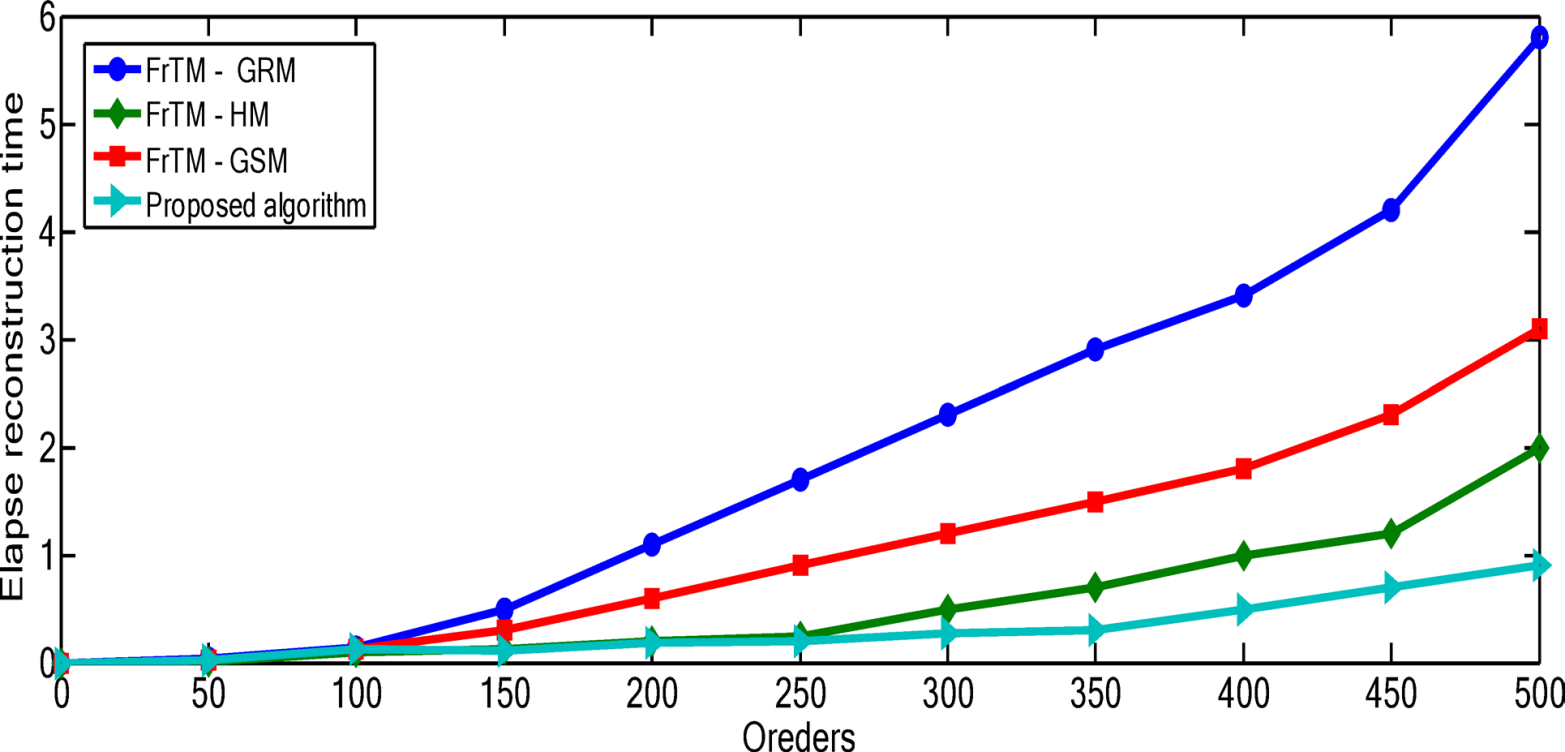
Elapse reconstruction time of the proposed approach and other traditional approaches: FrTMs- GSM, FrTMs – HM, and FrTMs – GRM at different orders.

**Fig. 14:**
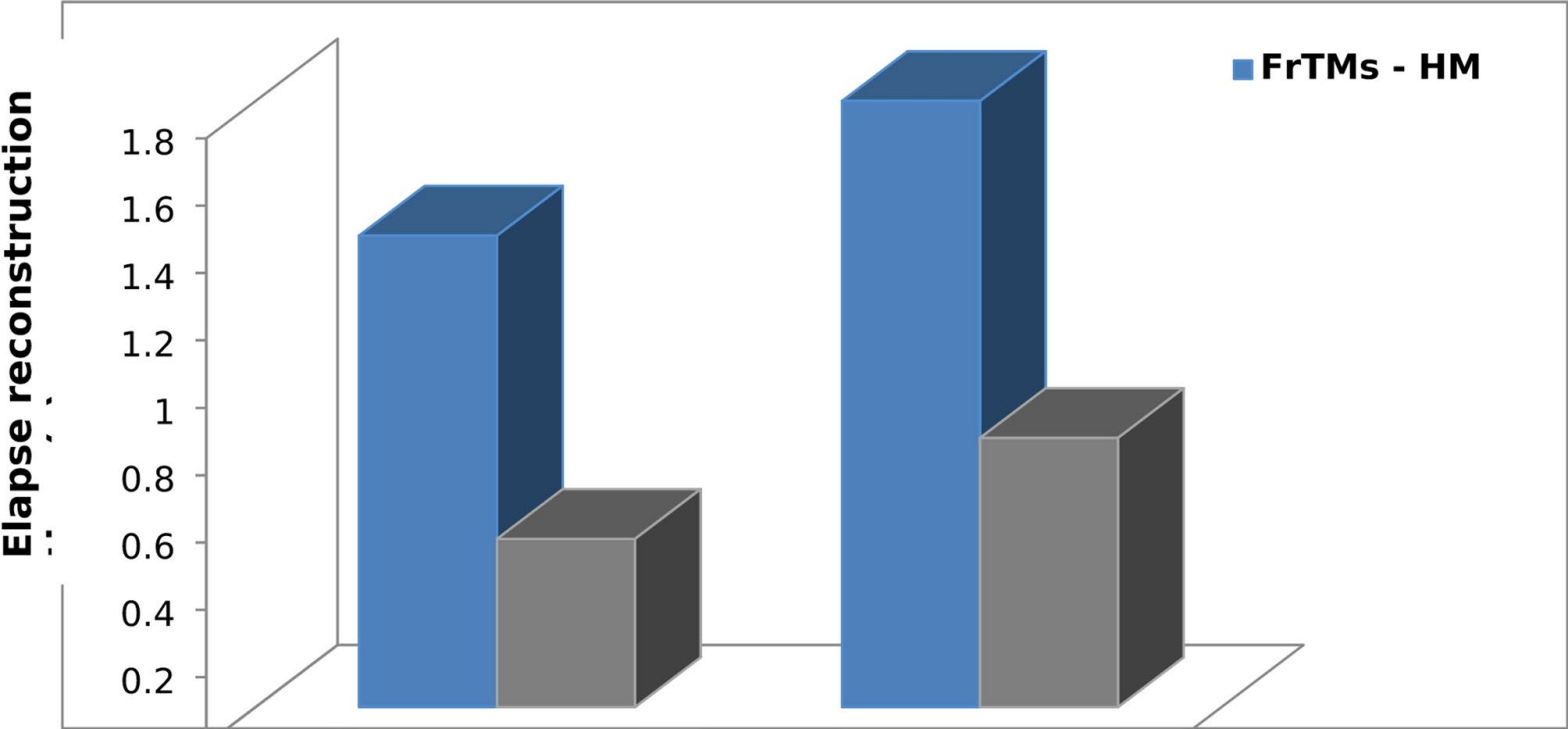
Elapsed reconstruction time of the reconstruction process for the proposed approach against FrTMs-HM using MIT-BIH Arrhythmia and CHB-MIT Scalp databases.

In order to assess the complexity of the algorithm under consideration, we conduct a comparative analysis of the elapsed reconstruction time between the proposed methods and three other methods, namely Fractional-order Generic Legendre Moments (FrTMs) (FrTMs-GSM, FrTMs-HM, and FrTMs-GRM). According to the findings presented in Fig. 13, it can be observed that the suggested method demonstrates superior performance compared to other methods in terms of elapsed reconstruction time across various orders. Visual representation in Fig. 13 shows the enhanced computational efficiency of our new approach compared to alternative methods (FrTMs with HM, GRM, and GSM implementations). A comprehensive temporal analysis in Table 7 contrasts the computational duration and efficiency metrics between our methodology and the FrTMs-HM approach. When processing MIT-BIH Arrhythmia Dataset signals, our algorithm achieves a mean reconstruction time of 0.5 s, significantly faster than the 1.4 s required by FrTMs-HM. Similarly, for CHB-MIT Scalp Dataset processing, our method requires only 0.8 s compared to 1.8 s for FrTMs-HM. This superior computational efficiency is further validated through the comparative metrics displayed in Table 8; Fig. 14.

**Table 7:** Comparing the proposed algorithm with existing techniques in watermarking.

Techniques	BER	PSNR	PRD
**Mathivanan et al. ** ^46^	**0.4522**	**58.60**	0.029
**N. Sharma et al. ** ^47^	**-**	**65.615**	0.291
**Khaldi et al. ** ^48^	**0.7812**	**48.380**	0.563
**Neeraj Kumar Sharm ** ^49^	**-**	**57.725**	0.271
**Natgunanathan et al. ** ^50^	**1.57**	**49.81**	-
**Proposed algorithm**	**0.25**	**66.847**	0.301

**Table 8:** Results of elapsed reconstruction time for the proposed method and FrTMs – HM on the two datasets.

	Average Elapse reconstruction time (s)		Efficiency Gain
	FrTMs - HM	Proposed algorithm	
MIT-BIH Arrhythmia Dataset	1.4	0.5	0.9
CHB-MIT Scalp Dataset	1.8	0.8	1

## Discussion

This study presents an improved reconstruction approach that utilizes FrTMs with the Schwarz-Rutishauser algorithm. In Quality and Performance Analysis, the proposed algorithm incorporating FrTMs with the Schwarz-Rutishauser method demonstrates superior performance compared to conventional approaches using FrTMs with GSM, HM, and GRM techniques. This superiority is evident across multiple evaluation metrics, particularly in reconstruction quality and computational efficiency. The exceptional performance can be attributed to two fundamental innovations: first, the implementation of normalized fractional-order Tchebichef moments with an optimized second-order recursive formula; and second, the application of the Schwarz-Rutishauser method for maintaining orthogonality while reducing computational complexity. Our experimental results demonstrate that the proposed algorithm achieves significantly higher reconstruction quality across both ECG and EEG signals compared to conventional approaches. For the MIT-BIH Arrhythmia dataset, our method achieves an average PSNR of 147.0814 dB and MSE of 0.0092, substantially outperforming alternative techniques. Similarly, for the CHB-MIT Scalp EEG dataset, the algorithm attains a PSNR of 118.449 dB and MSE of 0.0379, maintaining its performance advantage. This exceptional reconstruction fidelity is particularly crucial in biomedical applications where signal integrity directly impacts diagnostic accuracy and clinical decision-making. The watermarking implementation demonstrates remarkable imperceptibility characteristics, as evidenced by the high PSNR value of 66.847 dB. This represents a substantial improvement over existing methodologies such as those proposed by Khaldi et al. (48.380 dB) and Natgunanathan et al. (49.81 dB). Higher PSNR values indicate that our watermarking technique introduces minimal perceptible distortion to the original signal, a critical requirement for biomedical applications where signal fidelity must be preserved for accurate diagnosis. The PRD metric of 0.301, while slightly higher than Mathivanan et al.‘s approach (0.029), still falls within an acceptable range that maintains clinical utility of the watermarked signals. The robustness of the watermarking technique is demonstrated through the remarkably low BER of 0.25, indicating superior watermark extraction reliability compared to competing methods. This represents a significant improvement over Mathivanan et al. (0.4522), Khaldi et al. (0.7812), and particularly Natgunanathan et al. (1.57). The lower BER indicates enhanced resistance to potential signal degradation that might occur during transmission, storage, or processing in telemedicine applications. This robustness is particularly valuable in healthcare Internet of Things (IoHT) environments where signals may undergo various transformations before reaching their destination. With respect to Computational Efficiency the proposed algorithm demonstrates remarkable computational efficiency, particularly in the context of time-sensitive applications. For the MIT-BIH Arrhythmia Dataset, our method achieves an average reconstruction time of 0.5 s, compared to 1.4 s required by FrGLMs-HM—representing a 64% reduction in processing time. Similarly, for the CHB-MIT Scalp Dataset, our approach requires only 0.8 s versus 1.8 s for FrGLMs-HM, a 56% improvement. This enhanced computational efficiency stems from the Schwarz-Rutishauser algorithm’s complexity of O(mn²), which offers an n-fold reduction compared to traditional QR decomposition techniques such as GSM, HM, and GRM. Such improvements in processing speed make the proposed algorithm particularly suitable for real-time monitoring systems and resource-constrained IoHT devices. A comprehensive evaluation across all performance metrics demonstrates that the proposed algorithm achieves a well-balanced performance profile. While some existing methods may excel in individual metrics, our approach consistently performs well across all evaluation criteria, suggesting a more robust and practical solution for real-world applications. The combination of high PSNR, low BER, and acceptable PRD indicates that the algorithm successfully addresses the fundamental challenges in biomedical signal processing: maintaining high fidelity of the host signal, ensuring reliable watermark extraction, and minimizing overall signal distortion all while optimizing computational efficiency.

Despite the demonstrated advantages of our proposed scheme, several limitations warrant consideration and provide directions for future research. The performance of the FrTMs approach is influenced by the fractional order parameter selection. Our current implementation requires manual tuning of these parameters for optimal performance. This introduces a level of subjectivity and may require domain expertise to achieve optimal results across varying signal characteristics. While our approach demonstrates significant computational efficiency improvements compared to traditional methods, the processing time still increases substantially with higher polynomial orders. This may present challenges when implementing the algorithm in extremely resource-constrained environments such as wearable healthcare devices with limited processing capabilities and battery life. The current watermarking implementation focuses on optimizing imperceptibility and robustness but does not thoroughly explore capacity limitations. There exists a fundamental trade-off between embedding capacity and maintaining signal fidelity that warrants further investigation, particularly for applications requiring higher payload embedding.

In Future work should focus on developing intelligent algorithms that automatically determine optimal fractional orders based on signal characteristics. Developing adaptive parameter selection strategies based on signal characteristics. Future implementations could improve watermarking capacity by adopting a region-of-interest approach that identifies and utilizes signal segments with higher information redundancy for watermark embedding. The algorithm should be extended to support simultaneous processing of multimodal biomedical signals, such as combined ECG-respiratory monitoring or EEG-EMG analysis. This would involve developing techniques for cross-modal feature extraction and representation using fractional moments, which could enhance diagnostic capabilities in telemedicine applications.

## Conclusion

In this study, we introduced an improved algorithm for the reconstruction and watermarking of diverse bio-signals, specifically ECG and EEG. Our approach utilized FrTMs with an innovative implementation of the Schwarz-Rutishauser method. We began by deriving a three-term recurrence formula in its second-order form, enabling rapid calculation of FrCM basis functions. This mathematical advancement significantly improved computational efficiency compared to traditional methods. The integration of the Schwarz-Rutishauser algorithm, an efficient variant of QR decomposition, proved superior to conventional approaches such as GSM, HM, and GRM. This method maintained exceptional numerical accuracy and stability, particularly when processing higher-order FrTMs, which is crucial for high-fidelity bio-signal processing. Experimental validation using two benchmark datasets—MIT-BIH arrhythmia for ECG signals and CHB-MIT Scalp EEG for EEG bio-signals—demonstrated the algorithm’s superiority across multiple performance metrics. For reconstruction, our method achieved a PSNR of 147.08 dB and MSE of 0.0092 for ECG signals, and a PSNR of 118.45 dB and MSE of 0.0379 for EEG signals, substantially outperforming existing techniques. The computational efficiency was equally impressive, with average reconstruction times of 0.5 s for ECG and 0.8 s for EEG signals, representing significant improvements over competing methods. In the domain of watermarking, our algorithm achieved remarkable results with a PSNR of 66.85 dB, BER of 0.25, and PRD of 0.301. These metrics reflect a well-balanced approach that successfully addresses the fundamental challenge in watermarking, maintaining signal fidelity while ensuring robust watermark embedding and extraction. The algorithm’s performance demonstrates significant advantages over previous methods in the field, particularly in maintaining high PSNR while achieving low bit error rates. The dual capabilities of our algorithm in both reconstruction and watermarking make it particularly valuable for secure telemedicine applications and IoHT implementations. Future research directions could explore the application of this algorithm to other types of biomedical signals, investigation of its performance under various noise conditions, and extension to real-time processing environments. Additionally, exploring the algorithm’s potential in multi-modal signal fusion and its integration with deep learning approaches for feature extraction could yield further advances in both reconstruction and watermarking domains.

## Data Availability

The datasets analyzed during the current study are available in the PhysioNet repository. The MIT-BIH Arrhythmia Database (https://www.physionet.org/content/mitdb/1.0.0/) and the CHB-MIT Scalp EEG Database (https://physionet.org/content/chbmit/1.0.0/) were used for this analysis.
